# Graft compatibility emerges from the balance between regeneration and immunity: lessons from plant-plant interactions

**DOI:** 10.3389/fpls.2026.1892179

**Published:** 2026-09-08

**Authors:** Kaili Mao, Ruiduo Han, Zefeng Chen, Yanhong Zhou, Hannah Rae Thomas

**Affiliations:** 1 Department of Horticulture, Zhejiang University, Hangzhou, China; 2 Key Laboratory of Horticultural Plants Growth and Development, Agricultural Ministry of China, Hangzhou, China; 3 Hainan Institute, Zhejiang University, Sanya, China; 4 Zhejiang Provincial Key Laboratory of Horticultural Crop Quality Improvement, Zhejiang University, Hangzhou, China

**Keywords:** graft compatibility, growth-defense models, parasitic plants, plant grafting, plant-plant interactions

## Abstract

Plant grafting is a significant horticultural technique that enables the combination of desirable traits such as enhanced resilience, disease resistance, and productivity. Despite its widespread application, the mechanisms underlying graft compatibility remain poorly understood. Because grafting is largely an anthropogenic process, plants are unlikely to have evolved mechanisms specifically to recognize graft partners. Here, we propose that graft compatibility is not controlled by a dedicated recognition system, but instead emerges from the balance between existing tissue regeneration and immune surveillance pathways that evolved in other plant-plant interactions. We synthesize evidence from inter-plant communication, parasitic interactions, and damage-associated molecular pattern signaling (DAMPs) to show that these systems converge on conserved mechanisms regulating non-self perception, tissue regeneration, and long-distance communication. This evolutionary framework explains diverse observations across graft biology and provides a foundation for developing strategies to expand graft compatibility across economically important crops.

## Introduction

Plants must employ a wide range of strategies to respond to their constantly changing environment. Much like animal systems, plants encounter a diverse array of friends and foes, and it is critical that complex surveillance systems are in place to identify foreign organisms as “non-self” (Janeway, 1992). One of the most well-studied areas of plant biology revolves around the response of plants to pathogens, which requires the elicitation of both robust, as well as, specific immune responses. However, plants also interact with other plants, where both immune and developmental processes must be integrated to determine if the neighboring plant is beneficial or deleterious. Thus, plant-plant interactions provide a unique opportunity to understand how plants perceive non-self and regulate the balance between growth and defense. One key example of plant-plant interaction is the infestation of hosts by parasitic plants, which leach water and nutrients (Sanabria et al., 2010). In this instance, host plants must fend off enemies within the same kingdom, thereby leading to the evolution of plant-plant immune surveillance. In addition to plant parasitism, plants interact with one another for various reasons, such as protecting against self-pollination (Takayama and Isogai, 2005) and allocating resources (Si et al., 2025).

One remarkable type of plant-plant interaction is plant grafting. The origins of plant grafting are ancient, thought to have originated in oak (*Quercus* spp.), where pressure over time can force two neighboring branches of the same species together (Mudge et al., 2009; Lev-Yadun and Sprugel, 2011; Bormann and Graham, 1959). Similarly, natural grafting of tree roots occurs in oak as well as yellow birch (*Betula alleghaniensis*), where plant density forces nearby roots to press against one another, exposing their vascular tissues (Mudge et al., 2009; Lev-Yadun and Sprugel, 2011; Bormann and Graham, 1959). While both instances are natural, evolved processes driven by the necessity of wound healing, humans have co-opted the regenerative properties of plants, making grafting a key tool in horticulture and forestry.

The apical portion of a graft is referred to as the scion, and the root system as the (root)stock. Today, grafting is an effective strategy used across a wide range of species, including trees (Habibi et al., 2022) and herbaceous crops (Nawaz et al., 2016), to enhance productivity and resilience by combining genetically distinct scions and rootstocks (Goldschmidt, 2014; Augstein and Melnyk, 2025). For example, grafting can be used to induce dwarfism to improve planting and harvest practices. The “M9” rootstock is one of the most commonly used dwarfing varieties in apple (*Malus domestica*), where auxin signaling underlies the reduced stature of the grafted scion (Li et al., 2024). Grafting can be a strategy to improve fruit production, such as through graft-induced vigor. In *Arabidopsis thaliana* and Tomato (*Solanum lycopersicum*), *msh1* mutant rootstocks have been shown to induce vigor via root-to-shoot-mobile small interfering RNA (siRNA), which regulate the methylation of phytohormone genes in the scion and subsequent progeny (Kundariya et al., 2020). Grafting is also used to improve disease resistance. Among many examples, the well-known use of the North American grape (*Vitis labrusca*) rootstocks prevented the complete destruction of European wine cultivars (*Vitis vinifera*) by the invasive phylloxera insect pathogen (Mudge et al., 2009). Lastly, grafting is a key technique to promote abiotic stress tolerance. Interspecies grafting of *S. peruvianum* or *S. habrochaite*s rootstocks with *S. lycopersicum* scions can be used to promote temperature-related stress tolerance (Venema et al., 2008; Lee et al., 2024). While grafting requires combining genetically distinct plants to achieve these processes, the mechanism that enables the joining of plant varieties, species, and sometimes even families remains poorly understood.

For two plants to be successfully grafted, substantial vascular connections must form between the scion and the rootstock (Thomas et al., 2022). These successful grafts are known as compatible (Thomas et al., 2023). Some incompatible grafts even survive for weeks or months despite failed vascular reconnections. This is known as delayed graft incompatibility (Moreno et al., 1993). Several types of graft incompatibility have been identified, including metabolic (Habibi et al., 2022), physiological (sometimes known as localized) (Duman et al., 2020), pathogen-induced (Moreno et al., 2008; Seemüller and Schneider, 2004), as well as genetic (Thomas et al., 2023, 2024). Incompatibility can present as a wide range of symptoms, but key features include cell death at the junction and failed vascular connectivity.

Despite its widespread agricultural importance, the genetic basis of graft compatibility has remained poorly understood. Graft combinations are typically only successful between closely related species, either intra-genus or intra-species (Mudge et al., 2009; Thomas et al., 2023; Wulf et al., 2020). Most research on compatibility has been conducted in woody perennial crops, where compatibility remains a major limitation in breeding, constraining the transfer of valuable traits across genetic boundaries and limiting production (Baron et al., 2019; Fujii and Nito, 1972; Herrero, 1951; Garner, 1970). For example, *Prunus* spp., like peach (*Prunus persica*) and plum (*Prunus domestica*), can be used as rootstocks for almond *(Prunus amygdalus*) (Lordan et al., 2019), apricot (*Prunus armeniaca*) (Reig et al., 2018), and Japanese plums (*Prunus salicina*) (Reig et al., 2019), however, numerous instances of incompatibility have been reported (Moreno et al., 1993; Thomas et al., 2023; Habibi et al., 2022; Moreno et al., 2008; Seemüller and Schneider, 2004; Febres et al., 2024). Recent work in *Arabidopsis* (Melnyk et al., 2018), tomato (Thomas et al., 2022, 2023), and woody crops has expanded our understanding of the genetic regulators of grafting. Despite this, much remains unknown about how plants detect graft partners and determine compatibility status.

**Figure 1:**
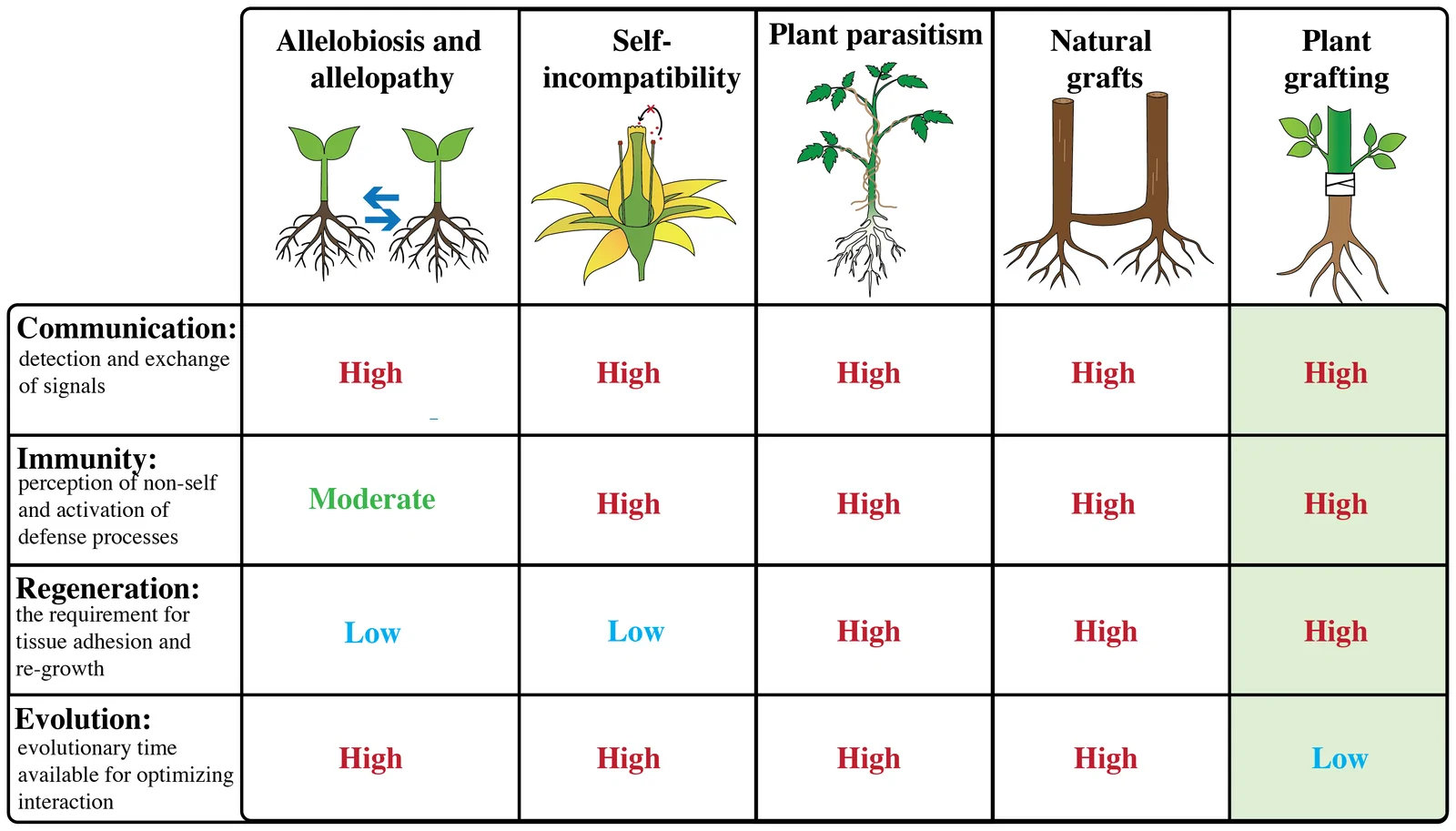
Evolutionary comparison of biological processes. Self‑/non‑self‑recognition systems recruit conserved biological modules, including communication, immunity, and regeneration, that have evolved and diversified over millions of years. The relative importance of each module (left panel) during allelobiosis and allelopathy, self-incompatibility, plant parasitism, natural grafting, and artificial plant grafting is summarized as high, moderate, or low. The final row indicates the relative degree of evolutionary optimization for each interaction, reflecting the amount of evolutionary time available for specialization. Plant grafting is highlighted in green.

One reason graft compatibility remains difficult to understand is that grafting itself is not an evolved biological process. Except for rare natural grafts, most graft combinations are anthropogenic associations between species that have never experienced any evolutionary selection for combined tissue regeneration. As a result, plants have not evolved a dedicated mechanism to recognize and respond to graft partners. Instead, grafting forces wounded tissues from genetically distinct lineages to respond using conserved signaling pathways, such as wound and defense responses. These pathways are evident across a wide range of plant-plant interactions (**Table 1**; **Figure 1**), where plants must exchange signals to communicate and respond appropriately. For example, parasitic plants can evade the host immune system to establish vascular connections, while hosts attempt to identify and prevent infection. Similarly, plants coordinate genetic outcomes by controlling pollen compatibility through self-incompatibility (SI). Although these interactions differ in their ecological functions, they repeatedly recruit common regulatory processes, including wound signaling, immune response, cell wall remodeling, vascular regeneration, and long-distance communication. Together, these processes reveal that plants possess a conserved molecular toolkit for interacting with non-self tissues and organisms that can be used to meet various environmental demands. Therefore, graft compatibility should be seen as an emergent property arising from the balance of two key processes in plant biology: immunity and regeneration. In this review, we use this evolutionary lens to examine how diverse plant-plant interactions inform our understanding of graft compatibility. By comparing these systems, we propose a unifying model in which graft compatibility emerges from distinct mechanisms that balance growth and defense.

**Table 1:** Comparison of conserved molecular pathways across plant–plant interactions relevant to graft compatibility.

Plant-plant interaction	Representative system	Signal / Receptor	Function	Relevance to graft compatibility	Evidence	Key references
Communication	Root exudates	Flavonoids, coumarins, benzoxazinoids, strigolactones	Inter-organism signaling and rhizosphere regulation	Rootstock-derived metabolites and microbial interactions influence graft performance and may regulate compatibility	✓	Marasco et al., 2018; Liu et al., 2018; Williams et al., 2021
	Mobile hormones	Auxin, ABA, JA, cytokinin, strigolactones	Coordinate long-distance communication between distant organs during development, wound repair, and environmental responses	Re-establishes vascular connections to allow rootstocks and scions to exchange information related to stress adaptation and defense responses after grafting	✓	Yang et al., 2022; Gasperini et al., 2015; Thomas et al., 2024
	Mobile proteins and peptides	Flowering locus T, CRISPR, HY5, CEPs, CLEs	Mobile proteins deliver long-distance signals involved in growth and development	Mobile signals transfer rootstock- or scion-derived traits and signals across the graft junction		Chen et al., 2016; Takahashi et al., 2018; Ota et al., 2020
	Mobile RNA	*BEL5*, *GAI*, sRNA	Phloem-mobile RNA potentially function as long-distance signals in diverse developmental processes			Banerjee et al., 2006; Haywood et al., 2005; Yoo et al., 2004
Immune surveillance PTI	Pathogen recognition	flg22/FLS2, chitin/CERK1, EF-Tu/EFR, LPS/LORE, etc.	Detection of non-self molecules and activation of conserved immune responses	Moderate PTI response promotes healing, whereas excessive activation may inhibit graft healing	✓	Han et al., 2026
	DAMP perception	OGs/Unknown, Pep1/PEPR, PSKs/PSKR, RALFs/FER	Links tissue damage with immune activation	DAMP signaling may determine the transition from defense to regeneration during graft healing		Yamaguchi et al., 2010; Harshith et al., 2024; Shen et al., 2025
	Plant parasitism	Crip21/CuRe1, Avr-Or7/HaOr7, Strigolactones/KAI22	Detection of parasitic plants	Demonstrates that plants recognize non-self plant tissues using extracellular immune receptors	✓	Hegenauer et al., 2020; Duriez et al., 2019; Park and Ronald, 2012; Takei et al., 2023
Immune surveillance ETI	Parasitic effectors	Unknown/RSG3-301, SHR4z/Unknown	Suppression of host immunity	Suggests successful vascular unions require immune suppression		Li and Timko, 2009; Su et al., 2020; Thomas et al., 2024
	Hybrid necrosis	DM11/DM10, Ne1/Ne2	Autoimmune activation between incompatible genotypes	Provides a possible model for genetic graft incompatibility during tomato-pepper grafting which activates broad NLR expression	✓	
Self recognition	Self-incompatibility	SRK/SCR, PrsS/PrpS, S-RNase/SLF	Recognition of self versus non-self pollens	Demonstrates that plants possess sophisticated recognition systems		Takasaki et al., 2000; Wheeler et al., 2010; Liu et al., 2014
Regeneration	Cell wall remodeling	GH9B3, EXG1, MYBs	Tissue adhesion and cell wall reconstruction	Plants have conserved regulators involved in cell-cell adhesion	✓	Notaguchi et al., 2020; Mazumdar et al., 2025; Yang et al., 2023a
	Hormones	Auxin, cytokinin, brassinosteroids, JA	Wound-induced regeneration	Plants have conserved regulators that coordinate vascular regeneration while balancing defense responses	✓	Serivichyaswat et al., 2024; Thomas et al., 2024
	Cambium-vascular development	TDIF–PXY–WOX4, VNDs	Cambial proliferation and vascular differentiation	Plant have a core conserved pathway that controls Cambium initiation during healing	✓	Hunziker and Greb, 2024; Kucukoglu et al., 2017; Kaga et al., 2020

Representative signaling pathways involved in plant communication, immune surveillance (pattern-trigger immunity; PTI and effector-triggered immunity; ETI), self-recognition (self-incompatibility), and regeneration (wound responsive homrones, cell wall remodeling, cambial regulators, and vascular regulators) are summarized alongside their proposed relevance to graft compatibility. “Representative system” lists major classes of interactions, “Signal/Receptor” provides representative ligand/receptors where relevant, “Evidence” indicates whether direct experimental evidence currently links the pathway to graft compatibility (✓, yes). Key references highlight representative studies supporting graft-related functions.

## Interplant communication predates plant grafting

**Figure 2:**
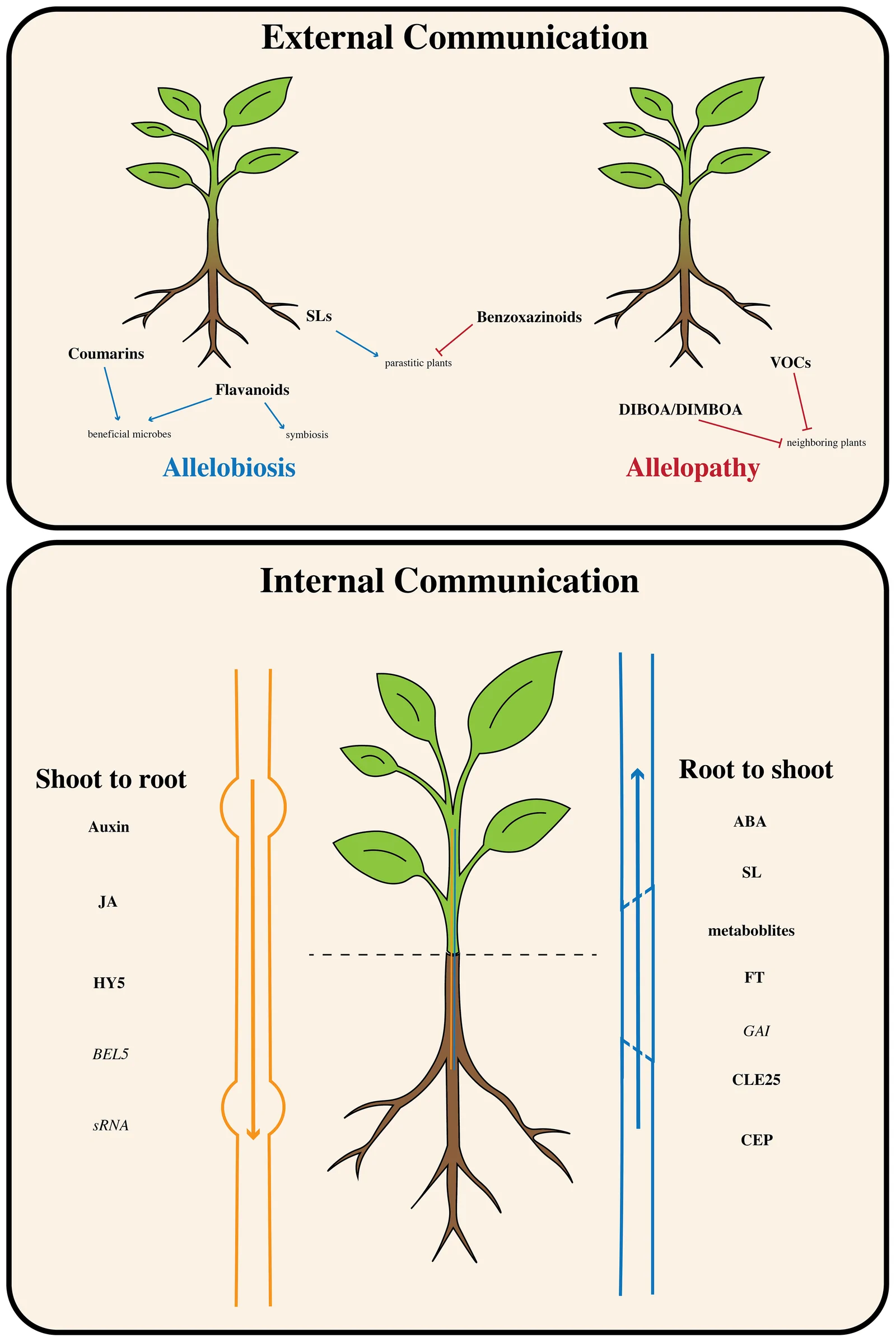
Ancient communication networks recruited during graft formation. (Top) Examples of external communication between plants and their environment through the release or perception of signaling molecules and root exudates. Allelobiosis (growth-promoting interactions; blue arrows) and allelopathy (growth-inhibiting interactions; red inhibitory lines) are illustrated around the root system. Representative signaling molecules associated with beneficial or inhibitory interactions are shown. (Bottom) Internal long-distance communication coordinates signaling between the shoot and root through the vascular system. Shoot-to-root transport occurs primarily through the phloem (orange), whereas root-to-shoot transport occurs predominantly through the xylem (blue)., with some molecules also transported through the phloem. Arrows indicate the predominant direction of long-distance transport, and the dashed line indicates the graft junction. Representative mobile molecules are shown. JA, jasmonic acid; ABA, abscisic acid; SL, strigalactone; FT, Flowering Locus T; VOCs, volatile organic compounds.

Plants rarely exist in isolation, so to facilitate interactions with neighboring plants, various forms of inter-plant communication have been established that allow stationary organisms to regulate their growth and defense by promoting or repressing nearby plants or by mediating their environment. Importantly, these methods of inter-plant communication evolved long before plant grafting. However, grafting utilizes these pre-existing systems during healing (**Figure 2**).

### External communication facilitates plant-plant interactions

One form of plant-plant communication involves the belowground environment, where plants release a wide array of compounds into the rhizosphere, known as root exudates. Root exudates have been shown to regulate the rhizosphere by affecting the chemical ecology of the surrounding soil, thus affecting other plants, microbes, and parasitic plants. Root exudate-mediated promotion of beneficial microbes, referred to as allelobiosis, has been extensively studied in the context of beneficial symbiotic interactions, such as those involving rhizobia and arbuscular mycorrhizal (AM) fungi, which are stimulated by plant-secreted flavonoids (Peters et al., 1986; Ruan et al., 2026) and strigolactones (SLs) (Soto-Cruz et al., 2021; Akiyama et al., 2010; Fiorilli et al., 2022). Secreted strigolactones (SLs) can also trigger germination of parasitic plants such as *Striga* spp. (Soto-Cruz et al., 2021; Yoneyama et al., 2010; Takei et al., 2023), while other compounds, such as coumarins (Steinauer et al., 2016), promote the proliferation of beneficial microbes while inhibiting pathogens (Feng et al., 2021; Huang et al., 2019; Hu et al., 2018; Stringlis et al., 2018). Through these interactions, the rhizome can also protect aerial plant parts from disease, a process known as induced systemic resistance (ISR) (Pieterse et al., 2014). The presence of certain root-associated mutualists, such as *Trichoderma* spp., can prime the plant immune system for subsequent pathogen infection, further underscoring the important role of the root system in plant health and protection (Walters et al., 2013). Together, these beneficial exudates can also promote beneficial plant-plant interactions including intercropping-induced resource allocation (Si et al., 2025) and floral timing cues (Stirnemann and Sasse, 2025).

Root exudates can also negatively affect nearby plants, a process known as allelopathy. These exudates may act to suppress seed germination or the growth of neighboring plants, thereby reducing resource competition (Sorty et al., 2025; Gattullo et al., 2018; Kegge et al., 2015) or preventing parasitic plant invasion (Cimmino et al., 2015; Soto-Cruz et al., 2021). Benzoxazinoids secreted by grasses are an example of phytotoxic root exudates (Schandry and Becker, 2020). In wheat relatives (*Triticeae* spp.), 4-dihydroxy-2*H*-1,4-benzoxazin-3(4*H*)-one (DIBOA) and 4-dihydroxy-*7-*methoxy-2*H*-1,4-benzoxazin-3(4*H*)-one (DIMBOA) exudates resulted in a significant decrease in *Sinapis alba* root growth (Belz and Hurle, 2005). Secreted volatiles also act broadly in the soil, such as the release of volatile jasmonates from wheat roots, which can be sensed by up to 100 other plant species (Kong et al., 2018).

Although root exudates do not directly mediate communication across graft junctions, the ecological principles governing rhizosphere communication remain highly relevant to grafting. Different rootstocks can influence the microbial populations, which in turn can affect plant growth, disease, and graft-induced vigor of the scion (Williams et al., 2021). For example, the tomato hybrid rootstock ‘Maxifort’ (*Solanum lycopersicum × S. habrochaites*) increases rhizosphere microbial diversity relative to self-grafted or non-grafted tomato plants (Grieneise et al., 2018). Similarly, in grafted grape and apple, rootstocks have been shown to influence microbial communities, with vigorous apple rootstocks promoting a more diverse bacterial microbiome than the dwarfing M9 (Liu et al., 2018), while grape rootstocks contained bacteria associated with growth-promoting traits (Marasco et al., 2018). These observations show that graft performance is influenced by interaction with the rhizosphere, in addition to communication between then scion and stock. Rather than representing a graft-specific phenomenon, rootstock-mediated modification of the soil highlights that grafted plants can utilize ancient communication networks that evolved for unrelated processes to improve graft performance.

### Internal communication crosses the graft junction

Following successful graft formation, vascular reconnection reestablishes a continuous stream of transport via phloem and xylem, linking distant tissues. Wounding, parasitism, and grafting all require the induction of these vascular reconstructions (Nishitani et al., 2002; Kirschner et al., 2023; Thomas et al., 2024), where hormones, metabolites, proteins, peptides, and RNAs move between distinct genetic partners, coordinating growth, development, and stress responses. While these mobile molecules may be essential for graft formation, they did not evolve to act on the graft process. Instead, they represent an endogenous communication system that normally connects the shoot and root systems.

Phytohormones provide clear examples of a conserved communication network. One of the most important regulators of plant regeneration is auxin (Asahina et al., 2011; Wang et al., 2014). Numerous studies have shown that removal of polar auxin transport is sufficient to interrupt both wound healing and graft reconnection (Asahina et al., 2011; Feng et al., 2024; Serivichyaswat et al., 2024).

Abscisic acid (ABA) serves as a major long-distance stress signal linking root perception of environmental conditions to shoot responses (Kuromori et al., 2022). Under drought conditions, ABA synthesized in roots is transported to aerial tissues where it promotes stomatal closure, modulates growth, and activates stress-responsive gene expression (Kuromori et al., 2022). Variation in rootstock-mediated ABA signaling has been associated with differences in drought tolerance and water-use efficiency in grafted crops, highlighting the importance of hormonal communication in environmental adaptation (Yang et al., 2022). SLs also function as long-distance signals from roots to shoots (Turnbull et al., 2002). Typically associated with their role in symbiosis and vegetative architecture, grafting wild-type rice rootstock onto a scion with a mutation in the SL biosynthetic gene *carotenoid cleavage dioxygenase 8* (*CCD8*) rescued the excessive tillering phenotype resulting from SL deficiency (Reeves et al., 2022; Chen et al., 2023).

Jasmonic acid (JA) and its derivatives accumulate rapidly following tissue damage (Kulkarni et al., 2024) and activate extensive transcriptional reprogramming associated with defense and stress adaptation (Howe and Jander, 2008; Glauser et al., 2008; Asahina et al., 2011). Perception of the bioactive conjugate jasmonoyl-isoleucine (JA-Ile) occurs through the F-box receptor CORONATINE INSENSITIVE1 (COI1), initiating signaling cascades that regulate defense, metabolism, and environmental responses (Yan et al., 2009). Jasmonates can move through vascular tissues and between adjacent cells, enabling local stress signals to generate systemic responses throughout the plant (Gasperini et al., 2015). Consistent with this role, grafting induces widespread changes in the expression of jasmonate-responsive genes, indicating that JA signaling is integrated into broader communication networks activated during graft establishment (Matsuoka et al., 2021; Asahina et al., 2011; Matsuoka et al., 2018). Evidence also suggests that jasmonate signaling may contribute to compatibility outcomes. In incompatible Solanaceous graft combinations, including tomato-pepper grafts, elevated JA signaling is accompanied by increased expression of defense-associated genes and accumulation of jasmonate-dependent metabolites such as steroidal glycoalkaloids (SGAs) (Bai et al., 2025; Thomas et al., 2024).

Likewise, numerous metabolites move through the vascular system. In grafted plants, these same molecules contribute after vascular reconnection, enabling the rootstock to influence the scion. The presence of graft-limiting metabolites has been hypothesized for decades (Mudge et al., 2009), but one of the most informative examples is seen in the quince-pear system. Quince (*Cydonia oblonga*) is commonly used as a dwarfing rootstock to pear (*Pyrus communis)* (Browning and Watkins, 1991). However, several economically valuable pear varieties, such as “Williams”, are graft-incompatible with quince (Tomaz et al., 2009; Herrero, 1951). This was attributed to high levels of the cyanogenic glucoside prunasin in quince rootstocks (Sánchez-Pérez et al., 2012). Because prunasin is phloem-mobile, it accumulates at the pear graft interface, where it is degraded by β-glucosidase and prunasin hydrolase, leaving behind the toxic byproduct, hydrogen cyanide (HCN) (Sánchez-Pérez et al., 2012), which leads to cell death and vascular degradation at the graft junction, making it a key example of metabolic incompatibility. Quince-pear incompatibility was successfully overcome using interstock grafting with the pear variety, “Old Home”, which contains the enzyme inhibitor capable of halting prunasin breakdown (Hudina et al., 2014). Other metabolic regulators of graft compatibility include phenolics, where some incompatible woody grafts have been associated with high levels of phenolic compounds (Mng'omba et al., 2008; Errea, 1998; Hudina et al., 2014). These examples demonstrates that graft compatibility may depend not only on the transport of specific metabolites but also on the capacity of recipient tissues to metabolize or detoxify them.

Beyond incompatibility, graft-mobile metabolites may contribute substantially to the desirable trait combinations sought in horticultural and perennial crop breeding programs. Rootstocks are known to influence fruit quality, stress tolerance, disease resistance, and overall productivity, yet the molecular basis of these effects remains poorly understood. Transport of secondary metabolites, intermediates, and other specialized compounds may alter the metabolic landscape of the scion and contribute to both resilience and productivity (Habibi et al., 2022; Dong et al., 2022). In woody perennial crops, where long-term yield and quality are major breeding goals, understanding how rootstocks regulate the movement and metabolism of graft-mobile compounds may prove essential for maximizing both compatibility and agronomic performance (La Malfa and Bennici, 2025). Furthermore, the identification of graft-mobile metabolites associated with incompatibility remains an underexplored area. Where extensive efforts in allelopathy research have employed bioassay-guided screens to identify phytotoxic root exudates (Bais et al., 2006), similar approaches could be applied to grafting systems to identify compounds that accumulate specifically in incompatible unions.

Recently, extensive research has been conducted on mobile proteins and RNAs. Hailing from the discovery of the mobile “florigen” (Chailakhyan, 1937), now known to be Flowering-Locus T (Wu et al., 2022), numerous mobile signals have been identified through grafting experiments. Shoot-to-root mobile HY5 (Chen et al., 2016) protein, CEP (Ota et al., 2020) and CLE (Takahashi et al., 2018) peptides, *BEL5* (Banerjee et al., 2006) and *GIBBERELLIC ACID INSENSITIVE* (*GAI)* (Haywood et al., 2005) mRNA, and high levels of small RNA (Yoo et al., 2004) have been identified, to name a few. Indeed, advancement in low-cost seqeucncing has led to the discovery of a high number of mobile RNAs and sRNAs between graft and parasitic-host partners (Li et al., 2021; Palauqui et al., 1997; Molnar et al., 2010).

A mechanism that parasitic plants and pathogens use to evade immune detection involves sRNAs, which regulate gene silencing by targeting mRNA for cleavage or epigenetic regulation. In tomato and *Arabidopsis*, infection with the fungus *Botrytis cinerea* led to the production of pathogenic sRNAs that could “hijack” the host AGONAUT1 (AGO1) protein and degrade host immune genes via RNA interference (RNAi) (Weiberg et al., 2013). Similarly, *Cuscuta campestris* was found to produce 22 nt microRNAs capable of targeting *Arabidopsis* mRNA, resulting in mRNA cleavage of genes involved in growth and defense, such as *BOTRYTIS-INDUCED KINASE 1 (BIK1)* (Lu et al., 2010). In contrast, host-induced gene silencing (HIGS) occurs when transgenic small interfering RNA (siRNA) is introduced into the host, targeting and degrading pathogen mRNA (Baulcombe, 2015).

Furthermore, efforts have been made to use grafting to introduce siRNA from the rootstock into the floral meristem. This technique allows grafting to serve as a molecular “vaccine,” delivering siRNA to existing plants via the phloem at the graft junction (Zhang et al., 2014). This same concept was famously utilized in 2023 to generate heritable transgene-free CRISPR Cas9-edited offspring via a graft-mobile editing system in *Arabidopsis* and *Brassica rapa* (Yang et al., 2023b). The area of mobile RNA has expanded to include the mechanisms that facilitate transport of RNAs and the potential involvement of extracellular vesicles (Baldrich et al., 2019; Shu et al., 2026). Notably, recent modeling has highlighted that the ability to confidently detect mobile RNAs using high-throughput sequencing alone requires careful consideration and, ideally, grafting evidence to definitively determine whether they have a functional role in plant physiology (Paajanen et al., 2025).

Collectively, mobile hormones, metabolites, proteins, and RNAs form a complex communication network through which rootstocks and scions can influence plant healing, physiology, and graft success. Conserved inter-plant communication proves the foundation for graft signaling. Long before modern humans, plants had already evolved sophisticated signaling systems to facilitate long-distance communication and interaction with neighboring plants as well as long distances through the vascular tissues. Thus, the molecules exchanged during grafting are not graft-specific. Rather, they reflect the recruitment of ancient signaling networks that are co-opted during grafting.

## Grafting activates immune processes

**Figure 3:**
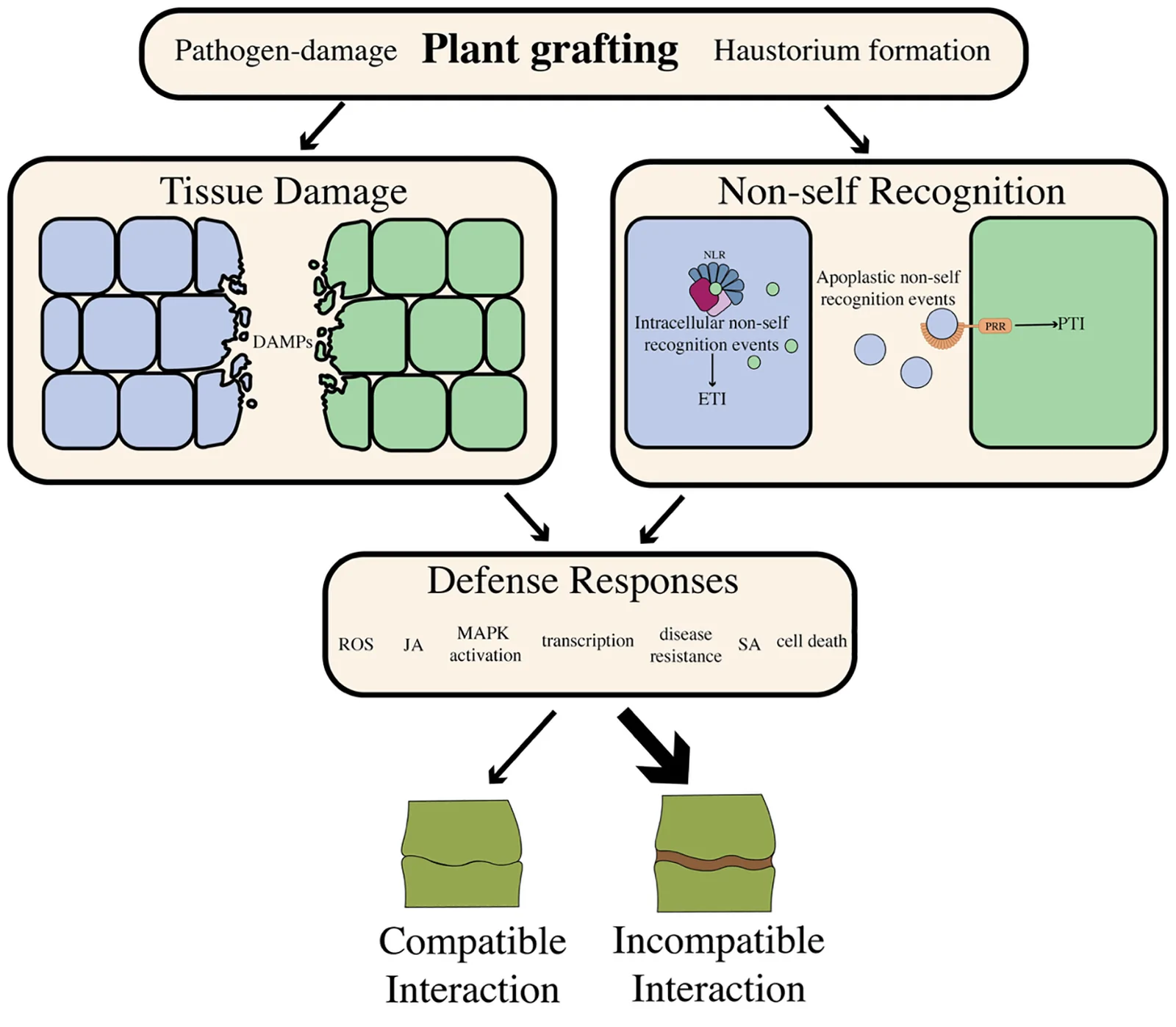
Conserved immune surveillance is activated during graft compatibility. Pathogen infection, parasitic plant invasion, and plant grafting activate conserved wound and immune signaling pathways. Tissue damage in two interacting plants (purple and green) releases cell wall fragments, intracellular molecules, and wound-induced peptides that function as damage-associated molecular patterns (DAMPs), endogenous signals released following cellular damage. DAMPs as well as any other apoplastic signals (purple circles) are perceived by pattern-recognition receptors (PRRs), leading to pattern-triggered immunity (PTI). Intracellular non-self molecules (green circles) may be recognized by nucleotide-binding leucine-rich repeat (NLR) immune receptors, activating effector-triggered immunity (ETI). Both immune pathways converge on defense responses including reactive oxygen species (ROS) production, mitogen-activated protein kinase (MAPK) signaling, jasmonic acid (JA) and salicylic acid (SA) signaling, transcriptional reprogramming, disease resistance, and cell death. When defense responses are transient or attenuated (thin arrow), regeneration proceeds and compatible grafts can form. In contrast, prolonged or excessive immune activation (thick arrow) suppresses regeneration and may result in graft incompatibility.

Plants have evolved multiple systems to distinguish self from non-self (Sanabria et al., 2010). These processes range from defense against pathogens to protection against self-fertilization. Although these systems evolved independently and perform distinct biological functions, they demonstrate that plants possess sophisticated molecular mechanisms for evaluating foreign biological material before permitting tissue integration. It has long been hypothesized that graft incompatibility results from immune responses (Yeoman, 1984; Kostoff, 1928). Successful grafting requires two genetically distinct plants to avoid excessive immune activation, despite a lack of evolutionary selection for this process. Because of this, plants must rely on existing surveillance systems that originally evolved to detect tissue damage, pathogen infection, and parasitic invasion (**Figure 3**).

Several types of incompatible grafting appear to fail due to excess immune response, such as pathogen-induced (virus-induced, bacterial-induced) (Moreno et al., 2008; Seemüller and Schneider, 2004; Febres et al., 2024) incompatibility and genetic incompatibility (Thomas et al., 2024). Both of these types of incompatibility activate immune-related processes such as excess cell death (Tedesco et al., 2023; Xiong et al., 2021; Febres et al., 2024; Thomas et al., 2024), increased ROS (Aloni et al., 2008; Nocito et al., 2010; Akiyama et al., 2010; Zarrouk et al., 2010; Hou et al., 2023), strengthened MAPK activation (Zhu et al., 2024; Wang et al., 2019b; Han et al., 2026), broad nucleotide-binding and leucine-rich repeat receptor (NLR) activation (Thomas et al., 2024), altered disease resistance (Liu et al., 2024; Han et al., 2026), and excess SA and JA (Cookson et al., 2014; Han et al., 2026) accumulation. Together, these symptoms suggest some cases of incompatibility are due to activated immune processes.

### Pattern-triggered immunity and non-self immunity

The first layer of defense during plant disease is pattern-triggered immunity (PTI), which comprises extracellular receptors known as pattern recognition receptors (PRRs) that detect microbe- or pathogen-associated molecular patterns (MAMPs or PAMPs), herbivore-associated molecular patterns (HAMPs), and damage-associated molecular patterns (DAMPs). When these molecules are bound by PRRs, a cascade of processes is activated, such as reactive oxygen species (ROS) burst, Ca^2+^ influx, the activation of mitogen-activated protein kinases (MAPK) cascades, and callose induction (Bigeard et al., 2015; Yu et al., 2024). Each PAMP has a main PRR that triggers downstream processes, such as the bacterial flagellin peptide flg22, which binds to FLAGELLIN SENSING2 (FLS2) (Chinchilla et al., 2006; Felix et al., 1999). PTI manifests as an activated immune system (Couto and Zipfel, 2016), which can induce increased resistance to a broad spectrum of subsequent infections (Zeidler et al., 2010; Wang et al., 2023).

The graft interface releases numerous wound signals that might trigger PTI. Since PTI responses have been observed during graft healing across multiple species, even compatible ones (Han et al., 2026), PTI does not appear to be inherently detrimental to graft formation. Problems likely arise when these defense processes become prolonged, preventing regeneration.

Parasitic plants, such as *Striga* spp., *Orobanche* spp., and *Cuscuta* spp., have evolved a structure known as a haustorium, capable of penetrating into the plant vascular system and extracting not only water and nutrients, but also transferring signaling molecules between the parasite and host through the haustoria (Kim and Westwood, 2015; Shen et al., 2023; Thomas et al., 2024). Most hosts fail to detect these invading parasites, making numerous crops susceptible to infestation. However, domesticated tomato is resistant to *Cuscuta reflexa* (Kaiser et al., 2015). During infestation, tomato elicits an immune response that induces the production of SA and JA (Runyon et al., 2010) and triggers hypersentitive response (HR) in the cells where haustoria have penetrated (Albert et al., 2004; Ihl. et al., 1988). It was later found that extracts from *C. reflexa* contained a glycine-rich cell wall peptide, coined Crip21 (Hegenauer et al., 2020), which could trigger typical PTI responses, such as a ROS burst, via a cell surface PRR, CUSCUTA RECEPTOR 1 (CuRe1) and the co-receptor SlSOBIR1 (Hegenauer et al., 2016). Thus making it clear that PRRs are also employed as receptors during plant-plant interactions (Hegenauer et al., 2016; Duriez et al., 2019).

In the *Orobanche cumana*-*Helianthus annuus* (sunflower) interaction, in which *O. cumana* penetrates the sunflower root to enter the vascular system, recent work has shown that resistance to *O. cumana* can be conferred by a member of the sunflower *Or* gene family, *HaOr7* (Duriez et al., 2019). Similar to PRRs, which perceive pathogens, HaOr7 is a LEUCINE-RICH REPEAT RECEPTOR-LIKE KINASE (LRR-RLK) and a predicted homolog of Xa21 in rice (Song et al., 1995). In rice, Xa21 is key for resistance to *Xanthomonas oryzae* pv. *Oryzae* (*Xoo*) by recognizing the bacterial peptide, Ax21 (Park and Ronald, 2012). Due to the sequence homology, HaOr7 is predicted to act as a PRR by binding to a hypothetical *O. cumana* peptide deemed Avr (Avirulence)-Or7 (Duriez et al., 2019).

In contrast to pathogen-derived molecules, it has also been shown that parasitic plants can also perceive host-derived molecular patterns as well. The quinone, 2,6-dimethoxy-1,4-benzoquinone (DMBQ), is perceived by the LRR-RLK, CANNOT RESPOND TO DMBQ 1 (CARD1) (Laohavisit et al., 2020). Unlike PTI, which induces anti-pathogenic processes, DMBQ perception by CARD1 in *Phtheirospermum japonicum* induced Ca^2+^-mediated haustorium induction. Interestingly, in non-parasitic *Arabidopsis*, DMBQ could also be perceived, leading to Ca^2+^ influx and MAPK induction, but not ROS, suggesting that quinone sensing is also involved in basal plant immunity, but does not overlap with PTI. Previous work identified quinones as stomatal regulators (Toh et al., 2018), and indeed, *card1* mutants were perturbed in stomatal immunity and more susceptible to *Pseudomonas syringae* pv. *tomato* DC3000 infection than wild-type (WT) (Laohavisit et al., 2020). These examples demonstrated the presence of plant PAMPs, suggesting that plants have likely evolved the ability to detect non-self plant interactions through cell-surface receptors, much as they detect microbes and insects.

### The role of effector trigger immunity on plant detection

The second layer of defense is ETI, where intracellular secreted pathogenic proteins, known as effectors, are sensed by intracellular NLRs. Effectors aim to evade the plant immune system and instead deactivate certain components of the immune response, making the plant susceptible to infection. Once perceived, NLRs can activate HR-related programmed cell death (PCD) in local tissue or trigger systemic acquired resistance (SAR)-mediated salicylic acid (SA) production systemically to prevent infection throughout the plant (Sanabria et al., 2010). ETI is hypothesized to be involved in immune-based graft incompatibility, especially tomato-pepper genetic incompatibility, since this graft combination elicits the upregulation of hundreds of NLRs in the heterografted rootstocks as well as the overaccumulation of SA (Thomas et al., 2024; Han et al., 2026).

Parasitic plants provide a useful evolutionary comparison to grafting because pathogen-host systems have undergone millions of years of adaptive coevolution in the effector-NLR arms-races. Evidence for parasitic plant effectors was first identified in *Striga*, an aggressive root parasite of the forage crop cowpea (*Vigna unguiculata* L. Walp.), where the resistant cultivar B301 (Timko et al., 2007) suddenly became susceptible to *Striga gesnerioides* race 4z (SG4z) (Huang et al., 2012). This phenomenon exemplified the gene-for-gene model underlying R gene evolution (Flor, 1971). Furthermore, it was later shown that B301 resistance to *S. gesnerioides* race 3 (SG3) was conferred by the NLR RSG3-301, although the effector remains unidentified (Li and Timko, 2009). Similarly, an SG4 effector protein, Suppressor of Host Resistance 4z (SHR4z), was identified as highly expressed in the haustorium and capable of attenuating the HR required for resistance (Su et al., 2020). Here, the effector was found to bind the cowpea ubiquitin E3 ligase VuPOB1, a positive regulator of HR (Su et al., 2020).

While PTI and ETI were originally considered pathogen-specific processes, it is now clear that plant PAMPs and effectors also exist. This supports the theory that graft compatibility is controlled by the growth-defense trade-off.

### Genetic incompatibility overlaps with hybrid necrosis

One theory for the cause of the over-active immune response seen in genetically incompatible grafts lies in hybrid necrosis (Hollingshead, 1930), which occurs when hybridized species have incompatible immune components, usually involving at least one NLR (Bomblies et al., 2007). Although observed in many plants, including *Crepis* spp. (Hollingshead, 1930), rice (*Oryza sativa*) (Yamamoto et al., 2010), *Nicotiana* (Yamada and MArubashi, 2003), wheat (*Triticum aestivum* L.) (Chu et al., 2006), and *Capsella* spp. (Sicard et al., 2015), hybrid necrosis was most clearly described in *Arabidopsis*, where crosses between Cdm-0 and other accessions, such as TueScha-9, led to severe necrosis in the cotyledon stage (Barragan et al., 2021). This instance of hybrid necrosis was found to be due to the presence of a truncated singleton NLR, DANGEROUS MIX 10 (DM10), in Cdm-0, which, when crossed with accessions containing DM11, triggered SA and JA production, upregulated hundreds of NLRs, and eventually led to cell death (Barragan et al., 2021). Since tomato-pepper incompatible grafts were also found to have upregulated SA and JA signaling, upregulated NLRs, and graft junction-specific cell death (Thomas et al., 2024), it is possible that hybrid necrosis underlies tomato-pepper genetic incompatibility. During grafting, tissues from two individuals are in such close proximity that there is a high chance of genetic exchange at the graft interface, and indeed, RNA (Thieme et al., 2015), DNA (Stegemann and Bock, 2009), extracellular circular DNA (Zhang et al., 2024), organelles (Hertle et al., 2021), and even entire nuclear genomes (Fuentes et al., 2014) can be horizontally transferred between cells at the graft interface. Therefore, similar mechanisms that control hybrid necrosis may also influence interspecies graft compatibility, in which components of separate immune systems, when mixed within a single cell, lead to cell death, thus blocking graft regeneration.

Grafting differs fundamentally from parasitic plant-host interactions because scions and rootstocks lack co-evolved mechanisms to recognize each other. Instead, immune activation during incompatible grafting is unlikely to be specific to grafting and instead reflects an evolutionary mismatch in which heterologous molecules or processes inadvertently exceed thresholds, triggering immune responses. PTI and ETI responses might occur because these conserved pathways mistakenly interpret foreign graft partners as indicators of pathogen or parasitic plant invasion. Because of this, numerous genetic mechanisms might lead to conserved immune-related phenotypes in diverse instances of graft incompatibility.

## Self-incompatibility as an example of non-self-detection

Self-incompatibility (SI), where plants can differentiate between self and non-self pollen, protects species against excessive inbreeding, and stands as a unique example of highly evolved non-self/self detection in plants. SI is largely controlled by one polymorphic locus called the S-locus (Takayama and Isogai, 2005). Through distinct molecular mechanisms, the S gene encodes alleles that regulate compatibility determination during pollen-pistil interaction. During Type 3 SI (*Papaveraceae*), the pollen S-gene (*P. rhoeas pollen S*: *PrpS*), and the stigma S-gene (*P. rhoeas stigma S*: *PrsS*), encodes a secreted protein. When SI occurs, it triggers Ca^2+^ influx and a cascade of responses resulting in PCD (Wheeler et al., 2009; Bosch and Franklin-Tong, 2008; Wheeler et al., 2010; Franklin-Tong et al., 2002; Chai et al., 2017; Wang et al., 2022). Similarly, during Type 4 SI (*Brassica*), the stigma S-locus receptor kinase (SRK) binds the pollen S-locus cysteine-rich protein (SCR) (Takayama et al., 2000; Takasaki et al., 2000). SI leads to the breakdown of fertilization-required compatibility factors and ROS production (Samuel et al., 2009; Sankaranarayanan et al., 2015; Scandola and Samuel, 2019). In *Arabidopsis*, this leads to autophagy of the pollen tube cell (Macgregor et al., 2022). In contrast, during Type 1 (*Solanaceae*) SI is controlled by a single pistil-expressed S-RNase and multiple pollen-expressed cytosolic S-loci F-boxes (SLFs) (Liu et al., 2014; Luu et al., 2000). S-RNAses are taken up into the pollen tube, where they bind to SLFs (Anderson et al., 1986). Non-self-recognition leads to the ubiquitination and degradation of the RNases, allowing pollen tube growth, whereas SI leads to RNA degradation of the pollen tube via the pistil RNase (Entani et al., 2014).

Parallels between pollen SI and the plant immune system have been previously drawn (Allen and Hiscock, 2008), as many SI systems are based on ligand-receptor pairings reminiscent of PAMP-PRRs. SI represents a key example of self and non-self detection that exists in the plant surveillance toolkit; however, it differs in that, with the exception of the development of the expanding pollen tube, SI lacks a significant regeneration mechanism.

## Damage-associated molecular patterns connect immunity and wound healing

The close relationship between immunity and regeneration is best illustrated during wound regeneration. Plants release a plethora of wound signals known as DAMPs into the apoplast during cellular compromise (Tanaka and Heil, 2021). Because of this, DAMPs act as immediate indicators of damage, regardless of whether it was caused by an innocuous mechanical wound or damage by a pathogen.

Like PAMPs, DAMPs are perceived by receptors and trigger additional immune responses. Most carbohydrates that act as DAMPs are components of the plant cell wall. These molecules are released into the apoplast during cellular degradation by pathogenic enzymes. Perhaps more relevant to grafting is the breakdown of homogalacturonan pectin into oligogalacturonides (OGs) by endogenous polygalacturonases, which occurs following wounding, as demonstrated in tomato leaves (Orozco-Cardenas and Ryan, 1999). OGs are one of the most extensively studied cell wall DAMP (Bishop et al., 1981; Davidsson et al., 2017). Treatment with OGs activates PTI processes such as MAPK activation, ROS burst, and resistance against *Botrytis* infection (Denoux et al., 2008; Galletti et al., 2011, 2008; Kohorn et al., 2009; Ferrari et al., 2013). Evidence first identified WALL-ASSOCIATED KINASE 1 and 2 (WAK1 and 2) as the proposed receptors due to their ability to bind OGs and trigger downstream responses (Brutus et al., 2010; Decreux et al., 2006; Kohorn et al., 2009), but recent work involving an *Arabidopsis* mutant lacking all five *WAK* genes was found to still execute immune responses when treated with exogenous OGs (Herold et al., 2024), demonstrating WAKs are not required for OG-induced PTI. Furthermore, studies have shown that WAKs function in coordination with other receptors, such as FERONIA (FER) (Dünser et al., 2019), which act as mechanical sensors of cell wall strain to elicit defense responses during wounding. Currently, the main OG receptor remains unclear.

During cell rupture, cytoplasmic contents also spill into the extracellular matrix. Extracellular ATP (eATP) is present at high concentrations within the cell, making it an excellent marker of compromised cellular integrity. Unlike OGs, which lack a clear functional receptor, P2 Receptor Kinase 1 and 2 (P2K1 and 2) have been validated as active receptors that trigger immune processes in response to high eATP (Choi et al., 2014; Pham et al., 2020; Tanaka et al., 2014; Jeter et al., 2004). Other cellular components, such as the amino acid glutamate, are released during wounding (Bellandi et al., 2022). Glutamate (glu) moves throughout the xylem and extracellular space, where it binds to and triggers the GLUTAMATE-LIKE RECEPTORS 3.3 (GLR) and GLR3.5 in *Arabidopsis*, which induce the intracellular influx of calcium (Toyota et al., 2018; Mousavi et al., 2013). The movement of glu in the plant was shown to trigger both the propagation of electrical signals via calcium and ROS waves. While *Arabidopsis* and tomato mutant rootstocks lacking long-distance electrical (*Atglr3.3glr3.6* or *Slglr3.3glr3.5*) and ROS (*AtrbohD* or *Slrboh1*) signals could still be successfully grafted onto WT scions (Zhan et al., 2025; Wang et al., 2019a), it is interesting to consider the role that these long-distance immune-mediating signals might play prior to or during compatibility determination.

Another nucleotide-based DAMP is extracellular DNA (eDNA), which can occur in the apoplast during necrotrophic infection and wounding. In pea (*Pisum sativum*), *Fusarium solani* produced a secreted DNase, which degraded host DNA (Klosterman et al., 2001). Fragmented DNA (less than 700 bp) could trigger responses, including Ca^2+^ influx, ROS, MAPK activation, and resistance to *Pseudomonas syringae* (Duran-Flores and Heil, 2018; Barbero et al., 2016). Interestingly, while all fragmented host DNA could elicit host immune responses, treatment with fragmented DNA from other species elicited a significantly attenuated response, suggesting that, however eDNA is surveilled, plants are capable of distinguishing self from non-self. It is worth noting that in a related process, *BREAST CANCER SUSCEPTIBILITY GENE 1* (*BRCA1*) and *BRCA1 ASSOCIATED RING DOMAIN PROTEIN* 1 (*BARD1*) homologs were identified as upregulated in incompatibly grafted tomato (Thomas et al., 2024). These genes are involved in DNA repair after genotoxic damage, suggesting that incompatibility may trigger DNA breakdown in tomato. It is possible that a molecular signal released by the opposite species or propagated by the host during failed healing (cell wall components or fragmented DNA) triggers many of the symptoms observed in genetic graft incompatibility (Thomas et al., 2023). To date, no published work has explored the role of OGs, eATP, glutamate, eDNA or other DAMPs during grafting, making this an important area for future studies.

Plants also secrete peptides during wound response. Peptide-DAMPs that have identified receptors include rapid alkalinization factors (RALFs), which bind to FERONIA (FER) (Stegmann et al., 2017; Pearce et al., 2001; Shen et al., 2025; Zhang et al., 2020); Plant elicitor peptide (PEPs), which bind to PLANT ELICITOR PEPTIDE RECEPTOR 1 (PEPR1) and PEPR2 (Huffaker et al., 2006; Yamaguchi et al., 2010, 2006); plasma membrane intrinsic proteins1/2 (PIP1/2), which binds to RECEPTOR-LIKE KINASES 7 (RLK7) (Hou et al., 2014); Systemin, which binds to SYSTEMIN RECEPTOR 1 (SYR1/2) (Pearce et al., 1991; Wang et al., 2018); serine-rich endogenous peptides 12 (SCOOP12), which binds to MALE DISCOVERER 1-INTERACTING RECEPTOR-LIKE KINASE 2 (MIK2) (Gully et al., 2019; Rhodes et al., 2021); and phytosulfokines (PSKs) that bind to PHYTOSULFOKINE RECEPTOR 1/2 (PSKR1/2) (Matsubayashi and Sakagami, 1996). Aside from species-specific DAMPs, most of these peptides are presumed to be secreted during grafting.

Recent work has suggested that PEPs and PSKs could be important to graft compatibility, as they actively regulate the balance of immunity and regeneration (Lori et al., 2015). PEPs are conserved species-specific DAMPs (Lori et al., 2015) that elicit PTI responses (Bartels et al., 2013). In contrast, PSKs, while first identified as wound-inducible, have since been shown to regulate growth and cell division (Kutschmar et al., 2009; Yang et al., 2000). It had previously been shown in tomato that PSK-regulated immunity to *Botrytis* was dependent on auxin signaling, further supporting PSK in the growth-defense nexus (Zhang et al., 2018). In rice (*Oryza sativa)*, wounding triggers expression of the OsPep3 precursor within 15 minutes. Wounding and Pep3 treatment induced rapid changes, including MAPK activation, JA signaling and defense genes such as *WRKY* TFs, as well as delayed expression of PSK precursors, especially OsPSK3 (Harshith et al., 2024). They then showed that the receptor, OsPSKR, associates with SOMATIC EMBRYOGENESIS RECEPTOR-LIKE KINASE 1 (SERK1), a homolog of *A. thaliana.* BRI1-ASSOCIATED RECEPTOR KINASE 1 (AtBAK1). *OsPSKR*-overexpression (OE) lines exhibited reduced wound response, whereas the *pskr* mutants displayed exaggerated and prolonged cell death, indicating that PSKR represses the late immune response to PEPs. The “constitutive cell death” phenotype seen in *pskr* is reminiscent of *Atbak1* mutants, in which loss of repression of immune processes led to autoimmune-induced cell death and hyperactivation of NLRs (Wu et al., 2020). Due to the striking similarity with genetic incompatibility (Thomas et al., 2024), it is curious to hypothesize that the antagonistic PEP-PSK interaction could regulate the delicate balance of immunity and regeneration critical to faucilaite graft compatibility. DAMP signaling is also critical for normal plant regeneration. Moderate activation can help coordinate wound repair and vascular regeneration, whereas excessive signaling might trigger immune processes that cause incompatibility. DAMPs are therefore an important bridge between immunity and regeneration.

## Graft compatibility requires tissue regeneration

**Figure 4:**
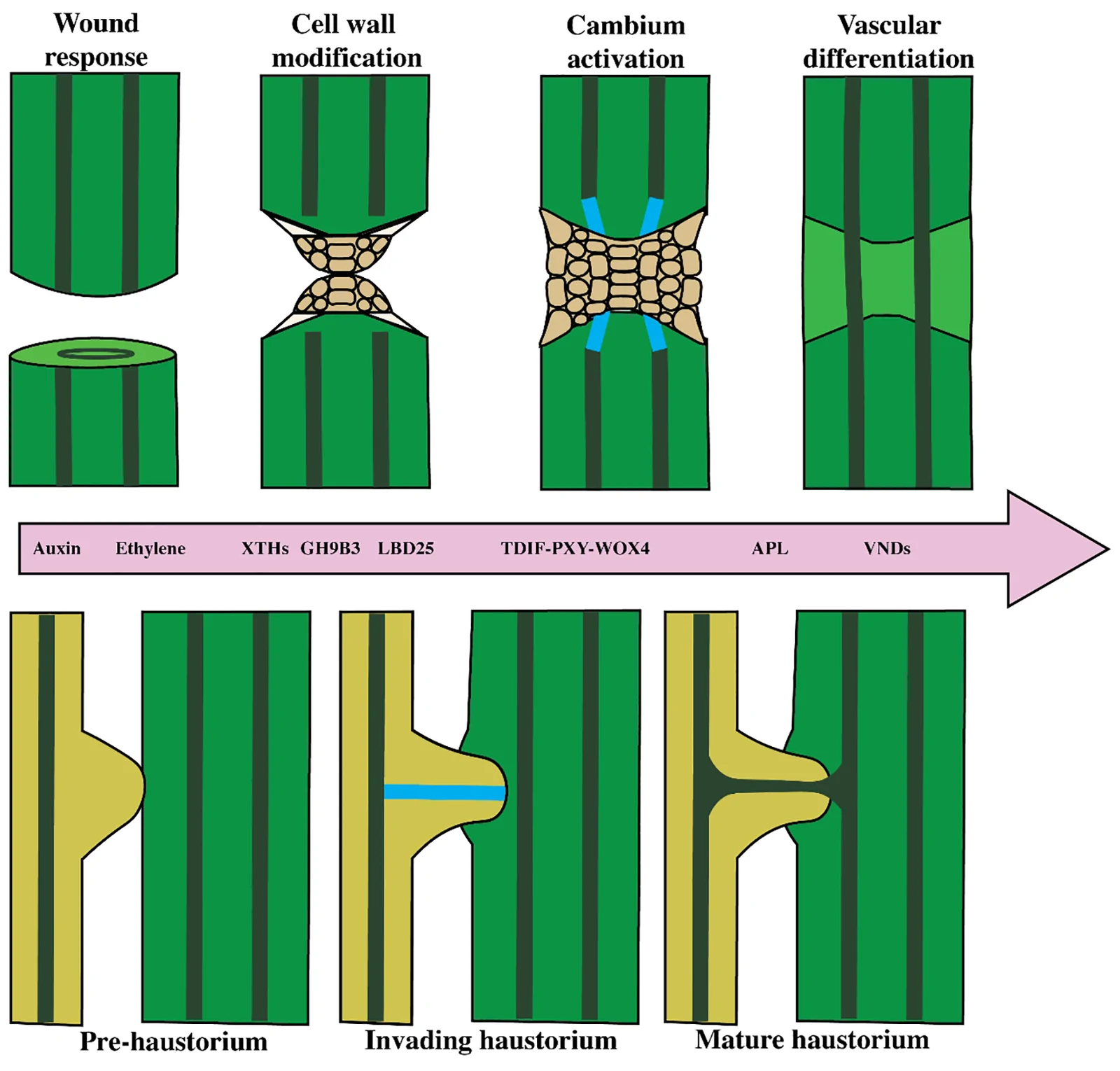
Shared regenerative programs utilized during graft healing and parasitic plant invasion. Comparison of tissue regeneration during plant graft healing (top) and parasitic haustorium development (bottom). During graft formation, regeneration progresses through four sequential stages: wound response, cell wall remodeling, cambial activation, and vascular differentiation. Callus tissue is shown in yellow, reactivated cambium in blue, and differentiated vascular tissue in dark green. Similarly, the parasitic haustorium (yellow) develops from a pre-haustorium into an invasive organ that penetrates host tissues before establishing vascular continuity with the host. Cambial activation is shown in blue and newly formed vascular connections in dark green. Representative regulatory pathways associated with each stage are shown along the timeline. The similarities between these developmental programs suggest that graft healing recruits ancient regeneration mechanisms that evolved during plant-plant interactions, particularly parasitic plant-host associations, to re-establish vascular connections between the scion and rootstock.

Compatible grafts must downregulate immunity, while promoting tissue regeneration, allowing for cell wall remodeling, cambial activation, and vascular redifferentiation. Several types of graft incompatibility revolve around failed regeneration. Physiological incompatibility, such as the requirement for etiolated grafted tissue in avocado or dormant scions in woody crops, tends to lack any immunity-centered phenotypes, but fail regardless due to a lack of regeneration (Duman et al., 2020). Similarly, metabolic incompatibility of pear-quince leads to the cellular breakdown of the phloem due to toxicity. Numerous cell wall- or vascular-regulating genetic mutants fail to heal due to disruption of key genes involved in regeneration (**Box 1**). The processes that underlie regeneration are therefore a critical component of graft compatibility. While other situations such as tissue culture provide the foundation of wound response biology, parasitic plants are an optimal system to examine how immunity and regeneration can be balanced to facilitate vascular reconnection. Grafted plants activate inherent wound response (Rasool et al., 2020) while parasitic plants form a haustorium (Kaiser et al., 2015; Kirschner et al., 2023; Shen et al., 2023), yet both require coordinated wound healing, cell wall remodeling, tissue adhesion, cambial activation and vascular differentiation (Hartmann et al., 2002; Kaga et al., 2020; Wakatake et al., 2018). These observations suggest that grafting and plant parasitism recruit a common developmental regeneration program (**Figure 4**).

Box 1 **Defining graft incompatibility.**

One of the major sources of confusion in the grafting literature is the interchangeable use of graft incompatibility and failed graft healing. Although both result in unsuccessful grafts, they arise from fundamentally different causes.

Failed graft healing refers to situations in which normal regenerative processes are experimentally disrupted. In these cases, successful grafting would otherwise be expected, but healing is prevented by mutations, transgenes, chemical treatments, or environmental perturbations that interfere with vascular regeneration. Examples include disruption of auxin transport, vascular differentiation, or cell wall remodeling genes. These experiments identify genes required for graft formation but do not necessarily reveal mechanisms of compatibility.

Graft incompatibility, by contrast, is an emergent property of the graft combination itself. Here, both partners retain the intrinsic capacity to heal wounds, yet successful vascular integration fails because inherent biological properties of one or both partners interfere with regeneration. These properties may include immune activation, metabolic incompatibility, developmental mismatch, or other genetically encoded traits. Thus, compatibility cannot be inferred simply from whether a gene is required for graft healing.

### Stage 1. Wound response and hormonal reprogramming

Following grafting and parasitic infection but preceding regeneration, the plant undergoes extensive hormonal fluctuation as part of the wound response. Auxin is one of the earliest and most important regulators of this transition (Wang et al., 2014). Auxin accumulates rapidly above the graft junction and functions to induce the differentiation of vascular tissue, cell proliferation, and tissue patterning. In *Arabidopsis*, blocking auxin signaling using the *bdl* (*Atiaa12*) mutant significantly reduced graft success in *Arabidopsis* (Serivichyaswat et al., 2024; Melnyk et al., 2015; Matsuoka et al., 2016). Apical auxin is required for the induction of *DNA BINDING WITH ONE FINGER* (*DOF*) transcription factors *HIGH CAMBIAL ACTIVITY2* (*HCA2*)*, TARGET OF MONOPTEROS6* (*TMO6*)*, DNA BINDING WITH ONE FINGER* (*DOF2.1*), and *DOF6* (Melnyk et al., 2018), and the direct target of DOF2.1, LONELY GUY4 (LOG4) (Smet et al., 2019), was also induced by grafting (Melnyk et al., 2018). *DOFs* are highly induced during *Arabidopsis* wounding and are required for vascular and non-vascular healing. It was shown that *DOF* induction also required cell wall damage, adding a second layer of input to necessitate expression (Melnyk et al., 2018; Zhang et al., 2022).

In parasitic plants, auxin preforms a similar function during haustoria formation. In *P. japonicum*, the perception of haustorium-inducing factors in the soil (such as plant exudates) leads to the induction of the auxin biosynthesis gene, *YUCCA3* (*YUC3*) (Ishida et al., 2016), a process that could be blocked in *Triphysaria versicolor* using the auxin transport inhibitor, triiodobenzoic acid (TIBA) (Tomilov et al., 2005). Since auxin is the key hormone required for vascular differentiation and reconnection, it is logical that similar processes are utilized during grafting as during other regenerative processes such as haustorium formation (Kaga et al., 2020; Wakatake et al., 2018), tissue culture (Hu et al., 2026), and bulbil formation (Shu et al., 2024). And indeed, we see auxin playing a central role to almost all regenerative process in plants.

Ethylene responses are also rapidly induced following tissue damage. *ERF114* and *ERF115* are induced during both wounding and graft healing of *Arabidopsis*, spruce, tomato, and pepper (Feng et al., 2024; Thomas et al., 2024; Zhang et al., 2022; Canher et al., 2022). Treating *Arabidopsis* with cell wall-modifying enzymes, such as cellulase and pectinase, was sufficient to activate *ERF115* expression in the stem, but, when combined with auxin, the effect was amplified (Zhang et al., 2022). Similarly, treatment with macerozyme, a pectinase, induced *ERF114* in the root (Canher et al., 2022). DNA damage to the root tip in the JA mutant, *coronatine insensitive1* (*coi1*), was able to show that wound-mediated *ERF115* expression did not require JA signaling, and while auxin was not required for *ERF115* induction following wounding, it was necessary for wound recovery (Canher et al., 2020). The authors then suggested *ERF114* and *115* could be induced by mechanical stress. The mechanosensory mutant, *feronia* (*fer*), which showed increased mechanical strain, in cooperation with auxin, was likely responsible for the induction of ERF114 and 115 (Canher et al., 2022).

Although its contribution appears more modulatory than essential, ethylene has been linked to tissue regeneration. Ethylene is induced and required for wound response (Li et al., 2018), but ethylene mutants in *Arabidopsis* (*ethylene insensitive 2*; *ein2*, *ethylene response 1*; *etr1*) appear to graft normally (Melnyk et al., 2015). This is contradicted in tobacco, where the application of an ethylene precursor (ACC) could enhance graft formation, whereas the ethylene biosynthesis inhibitor (AVG) delayed healing (Zhai et al., 2021). However, in *Arabidopsis*, the *ctr1* mutant, which over-accumulates ethylene, showed reduced phloem connectivity (Melnyk et al., 2015). Unlike the somewhat ambiguous effect of ethylene on graft healing, ethylene appears to positively regulate haustorium formation. In *P. japonicum* infection, mutations to *EIN2* and *ETR1*, as well as the ethylene signaling inhibitor, AgNO_3_, all resulted in severely compromised infection rates (Cui et al., 2020). Together, these observations suggest that auxin and ethylene provide the primary developmental framework for regeneration.

Jasmonates are also involved in localized tissue healing. In *Arabidopsis*, partial wounding to the floral hypocotyl led to high auxin/low JA above the wound, and low auxin/high JA below (Asahina et al., 2011). While JA is critical for healing and is indeed upregulated during grafting in *Arabidopsis*, the JA-biosynthesis mutant, *allene oxide synthase* (*aos*), was still capable of grafting, suggesting the exact role of JA during graft healing is more complex than wounding alone (Matsuoka et al., 2018; Kong et al., 2018). Regardless, it has been shown that JA is graft-mobile (Kong et al., 2018) and present in the cambium (Sehr et al., 2010), making it highly relevant to graft healing. In contrast, jasmonates tend to be active defensively in the host plant to protect against parasitic invasion (Dos Santos et al., 2003; Bar-Nun and Mayer, 2008), drawing another interconnected link between immunity and regeneration.

### Stage 2. Cell wall remodeling

Hormonal reprogramming alone is insufficient for successful vascular reconnection. Before vascular tissues can reconnect, the genetically distinct plants must first establish stable physical adhesion across the graft interface. This requires coordinated extracellular matrix remodeling, representing one of the earliest potential barriers to compatibility.

Some of the earliest transcriptional responses during graft healing are attributed to generalized wound responses. *ENHANCED XYLEM AND GRAFTING 1* (*EXG1*) and its possible interactor *RECEPTOR-LIKE PROTEIN 44* (*RLP44*) are rapidly induced by nematode infection, grafting, and infection with *Agrobacterium* and are negative regulators of vascular tissue regeneration (Mazumdar et al., 2025). Although the authors did not identify the elicitors for this, they hypothesized that hormones, ROS, changes to the cell wall, or turgor pressure may activate this gene, making it one of the first cell-wall-modifying genes expressed.

Among the first regulators identified during *Arabidopsis* wound healing were *ANAC071*, *ANAC096, *and *RAP2.6L. ANAC071*/*096* are highly expressed above the wound and *RAP2.6L* below (Asahina et al., 2011; Matsuoka et al., 2021). This was later found to be due to spatial accumulation of phytohormones, in which high auxin and ethylene promote *ANAC071*, and low auxin and high JA induce *RAP2.6L*. While *RAP2.6L* and JA were induced during grafting, they were later shown not to be required for healing (Matsuoka et al., 2018). In contrast, *ANAC071*/*096* were found to play a critical role in grafting. Auxin-induced *ANAC071* and *ANAC096* positively regulate proliferation during grafting, and *anac071anac096* double mutants display poor graft healing (Matsuoka et al., 2016; Zhang et al., 2022; Matsuoka et al., 2021).

ANAC071 was also shown to regulate xloglucan endo-transglycosylases (XETs) encoded by *xyloglucan endo-transglycosylases*/*hydrolase* (*XTH*) genes *XTH19* and *XTH20*, which are required for non-vascular cell differentiation in wounded stems (Pitaksaringkarn et al., 2014). XTH/XETs loosen and reorganize cell walls, facilitating adhesion between neighboring tissues. The *XTH* gene family has been validated as broadly upregulated during melon (*Cucumis melo*) grafting, especially during compatible grafting. A *CmXTH9* knockdown and an *Arabidopsis xth4xth7* double mutant both showed reduced graft healing and decreased callus proliferation (Xiong et al., 2026). In tomato, *XTHs* were differentially expressed in compatible and incompatible grafts, while gene regulatory networks based on the first week of compatible healing predicted *XTH16* to be regulated by *TOMATO HOMEOBOX GENE 1* (*THOM1*), and *SlXTH6* was predicted to be co-regulated by *JASMONATE-RESPONSIVE ERF 4* (*JRE4*) and *ETHYLENE RESPONSE FACTOR* (*ERF4*) (Thomas et al., 2022). In pepper grafts, the hub genes *LATERAL ORGAN BOUNDARIES DOMAIN 4* (*LBD4*), *MYB86*, *NGATHALIKE 1* (*NGAL1-like*), and two *ERFs* were all predicted to co-regulate *XTH22* and *XTH38*. Similarly, *XTHs* have been implicated in woody graft healing. In poplar-willow (*Salix rehderiana)* heterografts, *SrXTH16*, *SrXTH17*, *SrXTH25*, *PcXTH22*, and *PcXTH17* were all highly expressed in surviving combinations, which the authors suggested might be key regulators of this inter-generic graft pair (Yang et al., 2023a).

*XTHs* have also been implicated in *Cuscuta* infection, with *CrXTH1* and *CrXTH2* upregulated in the developing haustoria (Olsen et al., 2016; Johnsen et al., 2015; Hozumi et al., 2017). Haustorium penetration is often associated with cell wall-related genes, so the involvement of *XTHs* is logical (Ranjan et al., 2014; Yang et al., 2015; Johnsen et al., 2015; Hozumi et al., 2017). Whether *CrXTH1* and *CrXTH2* are more critical for remodeling of the host or parasite cell wall remains unclear.

Cellulose remodeling represents another critical component of tissue integration. Secreted glucanases degrade cellulose in the apoplast, perhaps enabling better contact between opposing tissues in the graft interface. *CELLULASE3* (*CEL3*)/*GLYCOSYL HYDROLASE9B3* (*GH9B3*), a β-1,4-glucanases has emerged as a determinant of interfamily graft compatibility in tobacco relative, *Nicotiana benthamiana* (Notaguchi et al., 2020) and *Petunia* (*Petunia hybrida*) (Kurotani et al., 2022). *N. benthamiana* is capable of forming graft adhesion with a diverse range of angiosperms potentially due to the activity of secreted *GH9B3*. Using *N. benthamiana* as an interstock, the authors were able to produce compatible tomato-*Arabidopsis* or tomato-*Chrysanthemum morifolium* grafts (Notaguchi et al., 2020).

Likewise, in *P. japonicum*, *PjGH9B3* was highly expressed during infection of *Arabidopsis* and during *P. japonicum-Arabidopsis* interspecies grafting (Kurotani et al., 2020). RNAi of *PjGH9B3* significantly reduced haustorium penetration, demonstrating that cell wall remodeling is critical for distant plant-plant regeneration. *LBDs* have also been identified as regulators of cell wall remodeling. *LBD25* was also identified as highly expressed in *C. campestris* haustorium (Jhu et al., 2021). In *Arabidopsis*, *LBD25* functions in auxin signaling during lateral root formation (Mangeon et al., 2011). Using HIGS in tomato, they showed that *CcLBD25* is critical for haustorium initiation, potentially through modification of the host cell wall (Jhu et al., 2021). In alignment with this, *LBD25* was among the most highly upregulated genes in the *Thesium chinense* haustorium, suggesting that this gene is activated in diverse species during infection (Ichihashi et al., 2018). The role of *LBD25* in reconnection is further supported by the identification of *SlLBD25* as a predicted hub gene during early tomato graft formation (Thomas et al., 2022).

Together, these transcription factors and enzymes appear to constitute a coordinated regeneration module responsible for preparing damaged tissues and altering existing cell walls prior to vascular regeneration. Notably, parasitic plants repeatedly recruit the same developmental module during host invasion, as those identified in grafted systems, suggesting that grafting has simply co-opted these generative pathways. Since adhesion is an initial step in regeneration, it stands to reason that genes that optimize early graft healing are often identified in graft studies as graft regulators (Box 1) and should be made targets for plant breeding to expand graft compatibility.

### Stage 3. Cambial Reactivation

Once physical adhesion has been established, regeneration depends upon the competency of *de novo* vascular formation. The need for cambial contact has long been noted in woody grafting handbooks (Hartmann et al., 2002), yet most mechanistic understanding of this process relies on *Arabidopsis* roots and hypocotyls, where vascular tissue development is initiated by the procambium. In contrast, grafting of woody crops occurs almost exclusively between mature stems containing an established secondary vascular cambium surrounded by highly lignified secondary xylem and bark (Baron et al., 2019). Woody grafting therefore requires not only activation of conserved developmental regulators but also reactivation of secondary cambial initials (Chen et al., 2019). Some grafted vegetative horticultural crops also face similar challenges, such as tomato, where woody secondary xylem, but no bark, is present at the time of grafting (Loupit et al., 2023). Despite these anatomical differences, remarkable conservation exists among the regulatory pathways initiating vascular regeneration. Cambial genes such as *PHLOEM INTERCALATED WITH XYLEM* (*PXY*)*, ARABIDOPSIS THALIANA HOMEOBOX FACTOR8* (*ATHB8*), *SUPPRESSOR OF MAX2 1-LIKE PROTEIN 5* (*SMXL5*), and *WUSCHEL RELATED HOMEOBOX4* (*WOX4*) are activated broadly during grafting (Thomas et al., 2022; Serivichyaswat et al., 2024). Similar developmental programs operate during parasitic infection, such as *C. campestris*, where transcriptional regulators of cambium activity in the haustorium were identified using RNA-seq (Kaga et al., 2020). Genes such as *CcMP* and *CcTMO5* were induced during the transition of search hyphae into xylem hyphae (Kaga et al., 2020).

One of the best-characterized regulators of cambial activity is the TRACHEARY ELEMENT DIFFERENTIATION INHIBITORY FACTOR (*TDIF*)*-PXY-WOX4* signaling module, present in diverse species such as herbaceous *Arabidopsis* (Hunziker and Greb, 2024), woody *Populus* (Kucukoglu et al., 2017; Etchells et al., 2015) and gymnosperms such as *Pinus* (Galibina et al., 2023). In *Arabidopsis*, TDIF CLAVATA3/EMBRYO SURROUNDING REGION RELATED 41 (CLE41) and CLE44 peptides produced in developing phloem activate PXY receptors within the procambium, promoting cambial proliferation through WOX4 while preventing premature xylem differentiation via the *GLYCOGEN SYNTHASE KINASE (*GSK3)-*BRI1-EMS-SUPPRESSOR (*BES1) pathway (Hunziker and Greb, 2024). Evidence from woody species demonstrates that this pathway extends well beyond primary vascular development. In *Populus*, *PttWOX4a/b* RNAi, reduced vascular cambium and secondary growth (Kucukoglu et al., 2017). Conversely, constitutive overexpression of *PttPXY* and *PttCLE41* resulted in vascular tissue abnormalities and poor plant growth (Kucukoglu et al., 2017; Etchells et al., 2015). Homologous signaling components have also been identified within the *C. campestris* and *C. japonica* haustoria, where *WOX4* and *PXY-like* were induced during the transition of search hyphae into xylem hyphae (Kaga et al., 2020) (Shimizu et al., 2018), while *PjWOX4* expression was also observed in *P. japonicum* haustorium using fluorescent markers (Wakatake et al., 2018). suggesting that parasitic vascular regeneration employs essentially the same developmental circuitry.

These findings are particularly important for grafting in woody perennial crops. Unlike herbaceous hypocotyl grafts, regeneration in mature stems must overcome lignification, dormancy, and the spatial organization of an already differentiated secondary cambium (Baron et al., 2019). Indeed, disorganized cambial tissue has been noted in several incompatible grafted tree combinations (Errea et al., 2001; Pina et al., 2012). Consequently, although the same molecular regulators appear conserved, their temporal activation and spatial regulation are likely to differ substantially between primary regeneration in herbaceous models and secondary cambial reactivation in woody species.

### Stage 4. Vascular redifferentiation

The final stage of regeneration is the differentiation of functional vascular tissues capable of restoring long-distance transport. Phloem reconnection occurs before the xylem, but remains comparatively less understood than xylem differentiation (Melnyk et al., 2015, 2018). In *Arabidopsis*, *ABERRANT LATERAL ROOT FORMATION 4* (*ALF4*), *AUXIN RESISTANT 1* (*AXR1*), and *HCA2* were first identified as key phloem regulators in the stock (Melnyk et al., 2015, 2018). However, *Arabidopsis* lines lacking auxin signaling in the promoter region of *ALTERED PHLOEM DEVELOPMENT* (*APL*), a phloem companion cell marker, showed a reduced, but non-significant reduction in phloem connectivity (Serivichyaswat et al., 2024). While not all parasitic plants form both xylem and phloem connections, *Orobanche* and *Cuscuta* spp. can (Dorr and Kollmann, 1995; Haupt et al., 2001; Aly et al., 2011). *C. japonica* expressed *CjAPL* and *CjSEOR1*, a sieve element marker gene, in the haustorium during infection, showing that similar genetic regulators control the haustorium phloem development (Shimizu et al., 2018).

As xylem connections formed between the *C. campestris* haustorium and the *Arabidopsis* host, the key xylem differentiation factor *VND7* (Kubo et al., 2005), along with known downstream genes *MYB46, MYB86, IRREGULAR XYLEM* (*IRX3*), and *IRX5* were upregulated (Kaga et al., 2020). *PjIRX3* was visualized in the haustorium using fluorescent markers, clearly building strong genetic parallels between the two parasitic plants (Wakatake et al., 2018). While *VNDs* are integral for xylem formation, single mutants fail to show phenotypes due to redundancy in the family (Gushino et al., 2024; Tan et al., 2018). Similarly, in tobacco, *Nbvnd7* mutants showed no effect on grafting, whereas inducible overexpression lines increased xylem formation in *Arabidopsis*-tobacco interspecies grafts (Huang et al., 2025). Completion of vascular differentiation marks the transition from wound healing to physiological integration, enabling long-distance transport of water, nutrients and signaling molecules between graft partners.

## The evolutionary context for graft compatibility

**Figure 5:**
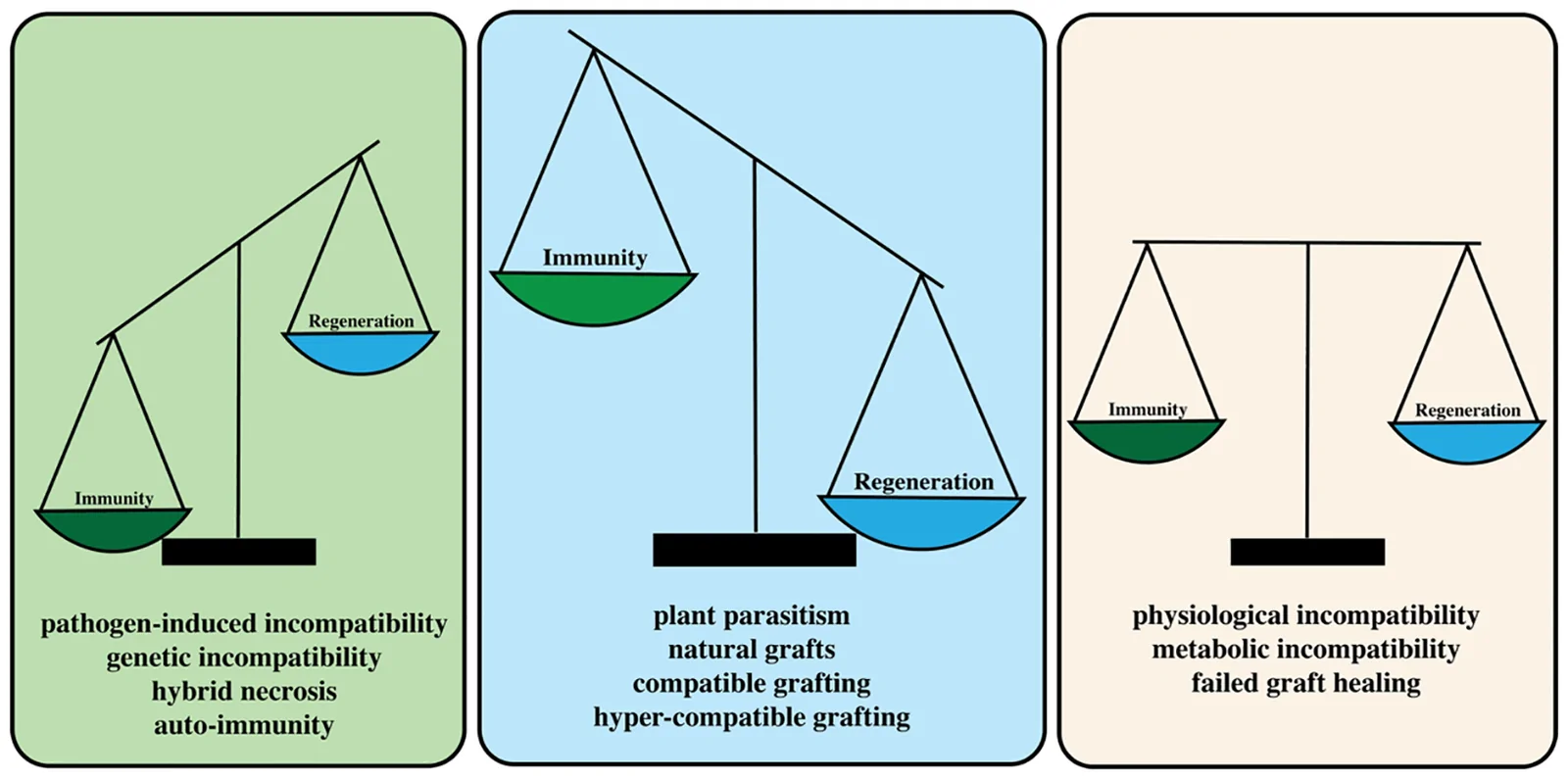
Graft compatibility emerges from the balance between regeneration and immunity. Conceptual model illustrating how the balance between immunity and regeneration determines plant-plant compatibility. (Left; green) Pathogen-induced incompatibility, genetic incompatibility, hybrid necrosis, and autoimmunity are characterized by excessive immune activation relative to regenerative capacity, resulting in tissue rejection, cell death, or failure of graft union formation. (Right; yellow) Physiological incompatibility, metabolic incompatibility, and failed graft healing represent situations in which regeneration is insufficient, despite relatively low immune activation, preventing successful vascular reconnection. These interactions illustrate that disruption of regenerative processes alone can lead to graft failure. (Middle; blue) Successful interactions, including plant parasitism, natural grafts, compatible grafting, and hyper-compatible grafting, require regeneration to be sufficiently robust to repair wounded tissues while preventing excess immune responses. Together, the relative balance between immunity and regeneration provides a conceptual framework for understanding compatibility across diverse plant-plant interactions.

Based on the evolution of plant-plant interactions, this review argues that graft compatibility should not be viewed as the consequence of isolated molecular pathways, but instead as a consequence of the balance between immunity and regeneration (**Figure 5**). Grafting therefore represents a novel context in which these conserved programs must operate despite lacking evolutionary optimization. During incompatibility, genetically distinct tissues experience evolutionary mismatch. This could be due to conserved processes such as PTI or ETI mistakenly interpreting wound-associated molecules as indicators of dangerous non-self (genetic incompatibility), or existing pathogens triggering defense processes (pathogen-induced incompatibility), causing defense processes to block regeneration. In contrast, incompatibility might be due to misfires in the regeneration process, where cell wall remodeling or cambium-vascular maintenance is perturbed (physiological or metabolic incompatibility). Similarly, mutagenesis of endogenous processes that lead to graft failure might be considered as a type of artificial graft compatibility. To date, most genes found to be required for graft healing are involved in scion-stock regeneration and would fall into this category.

In contrast, natural and compatible grafts are an example where the ability to regenerate has superseded any immunological restrictions. Plant parasitism is a long-term co-evolved process between the host and the pathogen and a key example of regeneration over immunity. In 1969, Kuijt referred to the haustorium as a “perfect graft” (Kuijt, 1969). At the mechanistic level, this is remarkably accurate, as both compatible grafts and haustoria converge on similar tasks: avoid the immune system and establish vascular connections. Successful parasitism therefore represents an evolved strategy for achieving “hyper-compatibility”. We propose a model in which graft compatibility depends on whether regenerative developmental programs can outcompete inadvertent immune activation. When this balance tips, different graft outcomes result.

This model can explain molecular observations related to graft compatibility. For example, GH9B3 may improve compatibility because it accelerates one of the earliest stages of regeneration (Notaguchi et al., 2020). Faster tissue adhesion might allow for regeneration to outpace immune signaling. Similarly, regulators of cambial activation and vascular differentiation, such as WOX4 (Thomas et al., 2022), may be key for compatibility because they shorten the period during which wounded tissues remain exposed to non-self surveillance. In contrast, activation of NLRs (Thomas et al., 2024) or other defense responses may lead to sustained defense responses that supersede regeneration. This model can also help to explain why more closely related plants are more likely to be graft compatible, because they experienced less evolutionary divergence in their immune-regeneration genetic modules.

## Implications for engineering broader graft compatibility

Viewing graft compatibility as a balance between regeneration and immunity has important implications for crop improvement. Traditional approaches have often focused on identifying individual compatibility genes or molecular markers associated with successful grafts. While the studies discussed in this review have identified numerous important regulators, these genes fail to fully explain why compatibility differs among species. Instead, engineering broader compatibility may require coordinated manipulation of both regenerative and immune pathways. Enhancing cell wall remodeling, vascular regeneration, or hormonal reprogramming alone may not be sufficient to improve graft outcomes if immune activation remains excessive. Likewise, suppressing immunity without maintaining regeneration may compromise wound healing or even increase susceptibility to pathogens.

Parasitic plants provide an evolutionary blueprint for overcoming this challenge. Rather than evolving entirely new developmental programs, they have progressively optimized the coordination between regeneration and immune regulation until tissue integration becomes highly reliable across diverse hosts. Understanding how evolution achieved this balance may therefore provide new strategies for extending graft compatibility beyond current taxonomic barriers.

## References

[B1] Akiyama K. Ogasawara S. Ito S. Hayashi H. (2010). Structural requirements of strigolactones for hyphal branching in AM fungi. Plant Cell Physiol. 51, 1104–1117. doi: 10.1093/pcp/pcq058 20418334 PMC2900820

[B2] Albert M. Werner M. Proksch P. Fry S. C. Kaldenhoff R. (2004). The cell wall-modifying xyloglucan endotransglycosylase/hydrolase LeXTH1 is expressed during the defence reaction of tomato against the plant parasite *Cuscuta reflexa*. Plant Biol. 6, 402–407. doi: 10.1055/s-2004-817959 15248122

[B3] Allen A. M. Hiscock S. J. (2008). Evolution and Phylogeny of Self-Incompatibility Systems in Angiosperms (Berlin, Heidelberg, GERMANY: Springer Berlin / Heidelberg).

[B4] Aloni B. Karni L. Deventurero G. Levin Z. Cohen R. Katzir N. . (2008). Physiological and biochemical changes at the rootstock-scion interface in graft combinations between *Cucurbita* rootstocks and a melon scion. J. Hortic. Sci. Biotechnol. 83, 777–783. doi: 10.1080/14620316.2008.1151246037339054

[B5] Aly R. Hamamouch N. Abu-Nassar J. Wolf S. Joel D. M. Eizenberg H. . (2011). Movement of protein and macromolecules between host plants and the parasitic weed *Phelipanche aEgyptiaca* Pers. Plant Cell Rep. 30, 2233–2241. doi: 10.1007/s00299-011-1128-5 21811827

[B6] Anderson M. A. Cornish E. C. Mau S. L. Williams E. G. Hoggart R. Atkinson A. . (1986). Cloning of cDNA for a stylar glycoproteinassociated with expression of self-incompatibility in *Nicotiana alata*. Nature 321, 38–44. doi: 10.1038/321038a0

[B7] Asahina M. Azuma K. Pitaksaringkarn W. Yamazaki T. Mitsuda N. Ohme-Takagi M. . (2011). Spatially selective hormonal control of RAP2.6L and ANAC071 transcription factors involved in tissue reunion in *Arabidopsis*. Proc. Natl. Acad. Sci. U.S.A. 108, 16128–16132. doi: 10.1073/pnas.1110443108 21911380 PMC3179063

[B8] Augstein F. Melnyk C. W. (2025). Modern and historical uses of plant grafting to engineer development, stress tolerance, chimeras, and hybrids. Plant J. 121, e70057. doi: 10.1111/tpj.70057 39982814 PMC11844807

[B9] Bai F. Wu M. Huang W. Xu W. Wang Y. Zhang Y. . (2025). Removal of toxic steroidal glycoalkaloids and bitterness in tomato is controlled by a complex epigenetic and genetic network. Sci. Adv. 11, eads9601. doi: 10.1126/sciadv.ads9601 39970214 PMC11837996

[B10] Bais H. P. Weir T. L. Perry L. G. Gilroy S. Vivanco J. M. (2006). The role of root exudates in rhizosphere interactions with plants and other organisms. Annu. Rev. Plant Biol. 57, 233–266. doi: 10.1146/annurev.arplant.57.032905.105159 16669762

[B11] Baldrich P. Rutter B. D. Karimi H. Z. Podicheti R. Meyers B. C. Innes R. W. (2019). Plant extracellular vesicles contain diverse small RNA species and are enriched in 10- to 17-nucleotide "Tiny" RNAs. Plant Cell 31, 315–324. doi: 10.1105/tpc.18.00872 30705133 PMC6447009

[B12] Banerjee A. Chatterjee M. Yu Y. Suh S. Miller W. Hannapel D. (2006). Dynamics of a mobile RNA of potato involved in a long-distance signaling pathway. Plant Cell 18, 3443–3457. doi: 10.1105/tpc.106.042473 17189340 PMC1785412

[B14] Barbero F. Guglielmotto M. Capuzzo A. Maffei M. E. (2016). Extracellular Self-DNA (esDNA), but not heterologous plant or insect DNA (etDNA), induces plasma membrane depolarization and calcium signaling in Lima Bean (*Phaseolus lunatus*) and Maize (*Zea mays*). Int. J. Mol. Sci. 17, 1659. doi: 10.3390/ijms17101659 27690017 PMC5085692

[B13] Bar-Nun N. Mayer A. M. (2008). Methyl jasmonate and methyl salicylate, but not cis-jasmone, evoke defenses against infection of *Arabidopsis thaliana* by *Orobanche aEgyptiaca*. Weed Biol. Manag 8, 91–96. doi: 10.1111/j.1445-6664.2008.00280.x40046247

[B15] Baron D. Esteves Amaro A. C. Pina A. Ferreira G. (2019). An overview of grafting re-establishment in woody fruit species. Sci. Hortic. 243, 84–91. doi: 10.1016/j.scienta.2018.08.01242574925

[B16] Barragan A. C. Collenberg M. Wang J. Lee R. R. Q. Cher W. Y. Rabanal F. A. . (2021). A truncated singleton NLR causes hybrid necrosis in *Arabidopsis thaliana*. Mol. Biol. Evol. 38, 557–574. doi: 10.1093/molbev/msaa245 32966577 PMC7826191

[B17] Bartels S. Lori M. Mbengue M. van Verk M. Klauser D. Hander T. . (2013). The family of Peps and their precursors in *Arabidopsis*: differential expression and localization but similar induction of pattern-triggered immune responses. J. Exp. Bot. 64, 5309–5321. doi: 10.1093/jxb/ert330 24151300

[B18] Baulcombe D. C. (2015). VIGS, HIGS and FIGS: small RNA silencing in the interactions of viruses or filamentous organisms with their plant hosts. Curr. Opin. Plant Biol. 26, 141–146. doi: 10.1016/j.pbi.2015.06.007 26247121

[B19] Bellandi A. Papp D. Breakspear A. Joyce J. Johnston M. G. de Keijzer J. . (2022). Diffusion and bulk flow of amino acids mediate calcium waves in plants. Sci. Adv. 8, eabo6693. doi: 10.1126/sciadv.abo6693 36269836 PMC9586480

[B20] Belz R. G. Hurle K. (2005). Differential exudation of two benzoxazinoids - One of the determining factors for seedling allelopathy of triticeae species. J. Agric. Food. Chem. 53, 250–261. doi: 10.1021/jf048434r 15656658

[B21] Bigeard J. Colcombet J. Hirt H. (2015). Signaling mechanisms in pattern-triggered immunity (PTI). Mol. Plant 8, 521–539. doi: 10.1016/j.molp.2014.12.022 25744358

[B22] Bishop P. D. Makus D. J. Pearce G. Ryan C. A. (1981). Proteinase inhibitor-inducing factor activity in tomato leaves resides in oligosaccharides enzymically released from cell walls. Proc. Natl. Acad. Sci. U.S.A. 78, 3536–3540. doi: 10.1073/pnas.78.6.3536 16593033 PMC319604

[B23] Bomblies K. Lempe J. Epple P. Warthmann N. Lanz C. Dangl J. L. . (2007). Autoimmune response as a mechanism for a dobzhansky-uuller-type incompatibility syndrome in plants. PloS Biol. 5, 1962–1972. doi: 10.1371/journal.pbio.0050236 17803357 PMC1964774

[B24] Bormann F. H. Graham B. F. Jr. (1959). The occurrence of natural root grafting in eastern white pine, *Pinus Strobus* L. and its ecological implications. Ecology 40, 677–691. doi: 10.2307/1929820

[B25] Bosch M. Franklin-Tong V. E. (2008). Self-incompatibility in *Papaver*: signalling to trigger PCD in incompatible pollen. J. Exp. Bot. 59, 481–490. doi: 10.1016/j.cub.2023.03.037 17872920

[B26] Browning G. Watkins R. (1991). Preliminary evaluation of new quince (*Cydonia oblonga* Miller) hybrid rootstocks for pears. J. Hortic. Sci. 66, 35–42. doi: 10.1080/00221589.1991.1151612237339054

[B27] Brutus A. Sicilia F. Macone A. Cervone F. De Lorenzo G. (2010). A domain swap approach reveals a role of the plant wall-associated kinase 1 (WAK1) as a receptor of oligogalacturonides. Proc. Natl. Acad. Sci. U.S.A. 107, 9452–9457. doi: 10.1073/pnas.1000675107 20439716 PMC2889104

[B28] Canher B. Heyman J. Savina M. Devendran A. Eekhout T. Vercauteren I. . (2020). Rocks in the auxin stream: Wound-induced auxin accumulation and ERF115 expression synergistically drive stem cell regeneration. Proc. Natl. Acad. Sci. U.S.A. 117, 16667–16677. doi: 10.1073/pnas.2006620117 32601177 PMC7368246

[B29] Canher B. Lanssens F. Zhang A. Bisht A. Mazumdar S. Heyman J. . (2022). The regeneration factors ERF114 and ERF115 regulate auxin-mediated lateral root development in response to mechanical cues. Mol. Plant 15, 1543–1557. doi: 10.1016/j.molp.2022.08.008 36030378

[B30] Chai L. Tudor R. L. Poulter N. S. Wilkins K. A. Eaves D. J. Franklin F. C. H. . (2017). MAP Kinase PrMPK9-1 contributes to the self-incompatibility response. Plant Physiol. 174, 1226–1237. doi: 10.1104/pp.17.00213 28385731 PMC5462039

[B31] Chailakhyan M. Kh. (1937) Hormonal Theory of Plant Development. Moscow: Akad. Nauk USSR. 198 pp.

[B33] Chen S. Song X. Zheng Q. Liu Y. Yu J. Zhou Y. . (2023). The transcription factor SPL13 mediates strigolactone suppression of shoot branching by inhibiting cytokinin synthesis in Solanum lycopersicum. J. Exp. Bot. 74, 5722–5735. doi: 10.1093/jxb/erad303 37504507 PMC10540736

[B32] Chen J. Wang L. Immanen J. Nieminen K. Spicer R. Helariutta Y. . (2019). Differential regulation of auxin and cytokinin during the secondary vascular tissue regeneration in *Populus* trees. New Phytol. 224, 188–201. doi: 10.1111/nph.16019 31230359

[B34] Chen X. Yao Q. Gao X. Jiang C. Harberd N. Fu X. (2016). Shoot-to-root mobile transcription factor HY5 coordinates plant carbon and nitrogen acquisition. Curr. Biol. 26, 640–646. doi: 10.1016/j.cub.2015.12.066 26877080

[B35] Chinchilla D. Bauer Z. Regenass M. Boller T. Felix G. (2006). The *Arabidopsis* receptor kinase FLS2 binds flg22 and determines the specificity of flagellin perception. Plant Cell 18, 465–476. doi: 10.1105/tpc.105.036574 PMC1356552 16377758

[B36] Choi J. Tanaka K. Cao Y. Qi Y. Qiu J. Liang Y. . (2014). Identification of a plant receptor for extracellular ATP. Science 343, 290–294. doi: 10.1126/science.343.6168.290 24436418

[B37] Chu C. G. Faris J. D. Friesen T. L. Xu S. S. (2006). Molecular mapping of hybrid necrosis genes Ne1 and Ne2 in hexaploid wheat using microsatellite markers. Theor. Appl. Genet. 112, 1374–1381. doi: 10.1007/s00122-006-0239-9 16518615

[B38] Cimmino A. Fernández-Aparicio M. Avolio F. Yoneyama K. Rubiales D. Evidente A. (2015). Ryecyanatines A and B and ryecarbonitrilines A and B, substituted cyanatophenol, cyanatobenzo[1,3]dioxole, and benzo[1,3]dioxolecarbonitriles from rye (*Secale cereale* L.) root exudates: Novel metabolites with allelopathic activity on *Orobanche* seed germination and radicle growth. Phytochemistry 109, 57–65. doi: 10.1016/j.phytochem.2014.10.034 25468713

[B39] Cookson S. J. Clemente Moreno M. J. Hevin C. Nyamba Mendome L. Z. Delrot S. Magnin N. . (2014). Heterografting with nonself rootstocks induces genes involved in stress responses at the graft interface when compared with autografted controls. J. Exp. Bot. 65, 2473–2481. doi: 10.1093/jxb/eru145 24692649 PMC4036518

[B40] Couto D. Zipfel C. (2016). Regulation of pattern recognition receptor signalling in plants. Nat. Rev. Immunol. 16, 537–552. doi: 10.1038/nri.2016.77 27477127

[B41] Cui S. Kubota T. Nishiyama T. Ishida J. K. Shigenobu S. Shibata T. F. . (2020). Ethylene signaling mediates host invasion by parasitic plants. Sci. Adv. 6, eabc2385. doi: 10.1126/sciadv.abc2385 33115743 PMC7608805

[B42] Davidsson P. Broberg M. Kariola T. Sipari N. Pirhonen M. Palva E. T. (2017). Short oligogalacturonides induce pathogen resistance-associated gene expression in *Arabidopsis thaliana*. BMC Plant Biol. 17, 19. doi: 10.1186/s12870-016-0959-1 28103793 PMC5248502

[B44] Decreux A. Thomas A. Spies B. Brasseur R. Van Cutsem P. Messiaen J. (2006). *In vitro* characterization of the homogalacturonan-binding domain of the wall-associated kinase WAK1 using site-directed mutagenesis. Phytochemistry 67, 1068–1079. doi: 10.1016/j.phytochem.2006.03.009 16631829

[B45] Denoux C. Galletti R. Mammarella N. Gopalan S. Werck D. De Lorenzo G. . (2008). Activation of defense response pathways by OGs and Flg22 elicitors in *Arabidopsis* seedlings. Mol. Plant 1, 423–445. doi: 10.1093/mp/ssn019 19825551 PMC2954645

[B46] Dong D. Shi Y. N. Mou Z. M. Chen S. Y. Zhao D. K. (2022). Grafting: a potential method to reveal the differential accumulation mechanism of secondary metabolites. Hortic. Res. 9, uhac050. doi: 10.1093/hr/uhac050 35591927 PMC9113227

[B47] Dorr I. Kollmann R. (1995). Symplasmic sieve element continuity between *Orobanche* and its host. Bot. Acta 108, 47–55. doi: 10.1111/j.1438-8677.1995.tb00830.x

[B48] Dos Santos C. V. Letousey P. Delavault P. Thalouarn P. (2003). Defense gene expression analysis of *Arabidopsis* thaliana parasitized by *Orobanche ramosa*. Phytopathology 93, 451–457. doi: 10.1094/phyto.2003.93.4.451 18944360

[B49] Duman Z. Hadas-Brandwein G. Eliyahu A. Belausov E. Abu-Abied M. Yeselson Y. . (2020). Short de-etiolation increases the rooting of VC801 avocado rootstock. Plants (Basel) 9. doi: 10.3390/plants9111481 33153170 PMC7693756

[B50] Dünser K. Gupta S. Herger A. Feraru M. I. Ringli C. Kleine-Vehn J. (2019). Extracellular matrix sensing by FERONIA and Leucine-Rich repeat extensins controls vacuolar expansion during cellular elongation in *Arabidopsis thaliana*. EMBO J. 38. doi: 10.15252/embj.2018100353 PMC6443208 30850388

[B51] Duran-Flores D. Heil M. (2018). Extracellular self-DNA as a damage-associated molecular pattern (DAMP) that triggers self-specific immunity induction in plants. Brain Behav. Immun. 72, 78–88. doi: 10.1016/j.bbi.2017.10.010 29042243

[B52] Duriez P. Vautrin S. Auriac M. C. Bazerque J. Boniface M. C. Callot C. . (2019). A receptor-like kinase enhances sunflower resistance to Orobanche cumana. Nat. Plants 5, 1211–1215. doi: 10.1038/s41477-019-0556-z 31819219

[B53] Entani T. Kubo K. Isogai S. Fukao Y. Shirakawa M. Isogai A. . (2014). Ubiquitin-proteasome-mediated degradation of S-RNase in a solanaceous cross-compatibility reaction. Plant J. 78, 1014–1021. doi: 10.1111/tpj.12528 24689760

[B54] Errea P. (1998). Implications of phenolic compounds in graft incompatibility in fruit tree species. Sci. Hortic. 74, 195–205. doi: 10.1016/s0304-4238(98)00087-9

[B55] Errea P. Garay L. Marín J. A. (2001). Early detection of graft incompatibility in apricot (Prunus Armeniaca) using *in vitro* techniques. Physiol. Plant 112, 135–141. doi: 10.1034/j.1399-3054.2001.1120118.x 11319025

[B56] Etchells J. P. Mishra L. S. Kumar M. Campbell L. Turner S. R. (2015). Wood formation in trees is increased by manipulating PXY-regulated cell division. Curr. Biol. 25, 1050–1055. doi: 10.1016/j.cub.2015.02.023 25866390 PMC4406943

[B57] Febres V. J. Fadli A. Meyering B. Yu F. Bowman K. D. Chaparro J. X. . (2024). Dissection of transcriptional events in graft incompatible reactions of "Bearss" lemon (Citrus limon) and "Valencia" sweet orange (C. sinensis) on a novel citrandarin (C. reticulata × Poncirus trifoliata) rootstock. Front. Plant Sci. 15, 1421734. doi: 10.3389/fpls.2024.1421734 38966146 PMC11222572

[B58] Felix G. Duran J. D. Volko S. Boller T. (1999). Plants have a sensitive perception system for the most conserved domain of bacterial flagellin. Plant J. 18, 265–276. doi: 10.1046/j.1365-313x.1999.00265.x 10377992

[B59] Feng H. Fu R. Hou X. Lv Y. Zhang N. Liu Y. . (2021). Chemotaxis of beneficial rhizobacteria to root exudates: the first step towards root-microbe rhizosphere interactions. Int. J. Mol. Sci. 22. doi: 10.3390/ijms22136655 34206311 PMC8269324

[B60] Feng M. Zhang A. Nguyen V. Bisht A. Almqvist C. De Veylder L. . (2024). A conserved graft formation process in Norway spruce and Arabidopsis identifies the PAT gene family as central regulators of wound healing. Nat. Plants 10, 53–65. doi: 10.1038/s41477-023-01568-w 38168607 PMC10808061

[B61] Ferrari S. Savatin D. V. Sicilia F. Gramegna G. Cervone F. Lorenzo G. D. (2013). Oligogalacturonides: plant damage-associated molecular patterns and regulators of growth and development. Front. Plant Sci. 4, 49. doi: 10.3389/fpls.2013.00049 23493833 PMC3595604

[B62] Fiorilli V. Forgia M. de Saint Germain A. D'Arrigo G. Cornu D. Le Bris P. . (2022). A structural homologue of the plant receptor D14 mediates responses to strigolactones in the fungal phytopathogen Cryphonectria parasitica. New Phytol. 234, 1003–1017. doi: 10.1111/nph.18013 35119708 PMC9306968

[B63] Flor H. H. (1971). Current status of the gene-for-gene concept. Annu. Rev. Plant Biol. 9, 275–296. doi: 10.1146/annurev.py.09.090171.00142341139587

[B64] Franklin-Tong V. E. Holdaway-Clarke T. L. Straatman K. R. Kunkel J. G. Hepler P. K. (2002). Involvement of extracellular calcium influx in the self-incompatibility response of Papaver rhoeas. Plant J. 29, 333–345. doi: 10.1046/j.1365-313x.2002.01219.x 11844110

[B65] Fuentes I. Stegemann S. Golczyk H. Karcher D. Bock R. (2014). Horizontal genome transfer as an asexual path to the formation of new species. Nature 511, 232–235. doi: 10.1038/nature13291 24909992

[B66] Fujii T. Nito N. (1972). Studies on the compatibility of grafting of fruit trees I. Callus fusion between rootstock and scion. J. Jpn. Soc Hortic. Sci. 41, 1–10. doi: 10.2503/jjshs.41.1

[B67] Galibina N. A. Moshchenskaya Y. L. Tarelkina T. V. Nikerova K. M. Korzhenevskii M. A. Serkova A. A. . (2023). Identification and expression profile of CLE41/44-PXY-WOX genes in adult trees Pinus sylvestris L. trunk tissues during cambial activity. Plants (Basel) 12. doi: 10.3390/plants12040835 36840180 PMC9961183

[B68] Galletti R. Denoux C. Gambetta S. Dewdney J. Ausubel F. M. De Lorenzo G. . (2008). The AtrbohD-mediated oxidative burst elicited by oligogalacturonides in Arabidopsis is dispensable for the activation of defense responses effective against Botrytis cinerea. Plant Physiol. 148, 1695–1706. doi: 10.1104/pp.108.127845 18790995 PMC2577270

[B69] Galletti R. Ferrari S. De Lorenzo G. (2011). Arabidopsis MPK3 and MPK6 play different roles in basal and oligogalacturonide- or flagellin-induced resistance against Botrytis cinerea. Plant Physiol. 157, 804–814. doi: 10.1104/pp.111.174003 21803860 PMC3192574

[B70] Garner R. J. (1970). The Grafter’s Handbook (London: Faber and Faber).

[B71] Gasperini D. Chauvin A. Acosta I. F. Kurenda A. Stolz S. Chételat A. . (2015). Axial and radial oxylipin transport. Plant Physiol. 169, 2244–2254. doi: 10.1104/pp.15.01104 26338953 PMC4634084

[B72] Gattullo C. E. Pii Y. Allegretta I. Medici L. Cesco S. Mimmo T. . (2018). Iron mobilization and mineralogical alterations induced by iron-deficient cucumber plants (Cucumis sativus L.) in a calcareous soil. Pedosphere 28, 59–69. doi: 10.1016/s1002-0160(15)60104-7

[B73] Glauser G. Grata E. Dubugnon L. Rudaz S. Farmer E. Wolfender J. (2008). Spatial and temporal dynamics of jasmonate synthesis and accumulation in Arabidopsis in response to wounding. J. Biol. Chem. 283, 16400–16407. doi: 10.1074/jbc.m801760200 18400744

[B74] Goldschmidt E. E. (2014). Plant grafting: new mechanisms, evolutionary implications. Front. Plant Sci. 5, 727. doi: 10.3389/fpls.2014.00727 25566298 PMC4269114

[B5555] Grieneisen M. L. Aegerter B. J. Scott Stoddard C. . (2018). Yield and fruit quality of grafted tomatoes, and their potential for soil fumigant use reduction. Agron. Sustain. Dev. 38, 29. doi: 10.1007/s13593-018-0507-5

[B75] Gully K. Pelletier S. Guillou M. C. Ferrand M. Aligon S. Pokotylo I. . (2019). The SCOOP12 peptide regulates defense response and root elongation in Arabidopsis thaliana. J. Exp. Bot. 70, 1349–1365. doi: 10.1093/jxb/ery454 30715439 PMC6382344

[B76] Gushino S. Tsai A. Y. Otani M. Demura T. Sawa S. (2024). VND genes redundantly regulate cell wall thickening during parasitic nematode infection. Plant Cell Physiol. 65, 1224–1230. doi: 10.1093/pcp/pcae048 38662403

[B77] Habibi F. Liu T. Folta K. Sarkhosh A. (2022). Physiological, biochemical, and molecular aspects of grafting in fruit trees. Hortic. Res. 9, uhac032. doi: 10.1093/hr/uhac032 35184166 PMC8976691

[B78] Han R. Meng K. Wang X. Xia X. Kang H. Zhou Y. . (2026). “ Salicylic acid mediates graft incompatibility by repressing auxin-dependent vascular reconnection in tomato,” in Biorxiv Preprint. 2026.07.30.741952. doi: 10.64898/2026.07.30.741952

[B79] Harshith C. Y. Pal A. Chakraborty M. Nair A. Raju S. Shivaprasad P. V. (2024). Wound-induced small-peptide-mediated signaling cascade, regulated by OsPSKR, dictates balance between growth and defense in rice. Cell Rep. 43, 114515. doi: 10.1016/j.celrep.2024.114515 39003743

[B80] Hartmann H. T. Kester D. E. Davies F. T. Geneve R. L. (2002). Plant Propagation: Principles and Practices (Upper Saddle River: Prentice Hall).

[B81] Haupt S. Oparka K. J. Sauer N. Neumann S. (2001). Macromolecular trafficking between Nicotiana tabacum and the holoparasite Cuscuta reflexa. J. Exp. Bot. 52, 173–177. doi: 10.1093/jxb/52.354.173 11181727

[B82] Haywood V. Yu T. Huang N. Lucas W. (2005). Phloem long-distance trafficking of Gibberellic acid-insensitive RNA regulates leaf development. Plant J. 42, 49–68. doi: 10.1111/j.1365-313X.2005.02351.x 15773853

[B83] Hegenauer V. Fürst U. Kaiser B. Smoker M. Zipfel C. Felix G. . (2016). Detection of the plant parasite Cuscuta reflexa by a tomato cell surface receptor. Science 353, 478–481. doi: 10.1126/science.aaf3919 27471302

[B84] Hegenauer V. Slaby P. Körner M. Bruckmüller J. A. Burggraf R. Albert I. . (2020). The tomato receptor CuRe1 senses a cell wall protein to identify Cuscuta as a pathogen. Nat. Commun. 11, 5299. doi: 10.1038/s41467-020-19147-4 33082345 PMC7576778

[B85] Herold L. Ordon J. Hua C. Kohorn B. D. Nürnberger T. DeFalco T. A. . (2024). Arabidopsis WALL-ASSOCIATED KINASES are not required for oligogalacturonide-induced signaling and immunity. Plant Cell 37, koae317. doi: 10.1101/2024.04.15.589471 39665686 PMC11684076

[B86] Herrero J. (1951). Studies of compatible and incompatible graft combinations with special reference to hardy fruit trees. J. Hortic. Sci. 26, 186–237. doi: 10.1080/00221589.1951.1151373637339054

[B87] Hertle A. P. Haberl B. Bock R. (2021). Horizontal genome transfer by cell-to-cell travel of whole organelles. Sci. Adv. 7, eabd8215. doi: 10.1126/sciadv.abd8215 33523859 PMC7775762

[B88] Hollingshead L. (1930). A lethal factor in crepis effective only in an interspecific hybrid. Genetics 15, 114–140. doi: 10.1093/genetics/15.2.114 17246594 PMC1201059

[B90] Hou Y. Qin X. Qiu H. Li D. Xu N. Zhang S. . (2023). Metabolite profiling and transcriptome analyses provide insight into the regulatory network of graft incompatibility in litchi. Front. Genet. 13, 2022. doi: 10.3389/fgene.2022.1059333 36685870 PMC9849251

[B89] Hou S. Wang X. Chen D. Yang X. Wang M. Turrà D. . (2014). The secreted peptide PIP1 amplifies immunity through receptor-like kinase 7. PloS Pathog. 10, e1004331. doi: 10.1371/journal.ppat.1004331 25188390 PMC4154866

[B91] Howe G. A. Jander G. (2008). Plant immunity to insect herbivores. Annu. Rev. Plant Biol. 59, 41–66. doi: 10.1146/annurev.arplant.59.032607.092825 18031220

[B92] Hozumi A. Bera S. Fujiwara D. Obayashi T. Yokoyama R. Nishitani K. . (2017). Arabinogalactan proteins accumulate in the cell walls of searching hyphae of the stem parasitic plants, Cuscuta campestris and Cuscuta japonica. Plant Cell Physiol. 58, 1868–1877. doi: 10.1093/pcp/pcx121 29016904

[B93] Hu L. F. Robert C. A. M. Cadot S. Zhang X. Ye M. Li B. B. . (2018). Root exudate metabolites drive plant-soil feedbacks on growth and defense by shaping the rhizosphere microbiota. Nat. Commun. 9, 2738. doi: 10.1038/s41467-018-05122-7 30013066 PMC6048113

[B94] Hu Z. Zhang L. Bie S. Niu J. Chen K. Ji X. . (2026). A CLE-RLK-LBD signaling module promotes de novo shoot regeneration in plants. J. Integr. Plant Biol. 00, 1–18. doi: 10.1111/jipb.70300 42186103

[B95] Huang A. C. C. Jiang T. Liu Y. X. Bai Y. C. Reed J. Qu B. Y. . (2019). A specialized metabolic network selectively modulates Arabidopsis root microbiota. Science 364, eaau6389. doi: 10.1126/science.aau6389 31073042

[B97] Huang K. Mellor K. E. Paul S. N. Lawson M. J. Mackey A. J. Timko M. P. (2012). Global changes in gene expression during compatible and incompatible interactions of cowpea (Vigna unguiculata L.) with the root parasitic angiosperm Striga gesnerioides. BMC Genomics 17, 402. doi: 10.1186/1471-2164-13-402 22900582 PMC3505475

[B96] Huang C. Toyokura K. Murakami E. I. Ishiwata A. Kurotani K. I. Notaguchi M. (2025). Nicotiana benthamiana VASCULAR-RELATED NAC-DOMAIN7-2 (NbVND7-2) has a role in xylem formation during interfamily grafting. J. Exp. Bot. 76, 2207–2221. doi: 10.1093/jxb/eraf074 PMC12116178 39987463

[B98] Hudina M. Orazem P. Jakopic J. Stampar F. (2014). The phenolic content and its involvement in the graft incompatibility process of various pear rootstocks (Pyrus communis L.). J. Exp. Bot. 171, 76–84. doi: 10.1016/j.jplph.2013.10.022 24484960

[B99] Huffaker A. Pearce G. Ryan C. A. (2006). An endogenous peptide signal in Arabidopsis activates components of the innate immune response. Proc. Natl. Acad. Sci. U.S.A. 103, 10098–10103. doi: 10.1073/pnas.0603727103 16785434 PMC1502512

[B100] Hunziker P. Greb T. (2024). Stem cells and differentiation in vascular tissues. Annu. Rev. Plant Biol. 75, 399–425. doi: 10.1146/annurev-arplant-070523-040525 38382908

[B101] Ichihashi Y. Kusano M. Kobayashi M. Suetsugu K. Yoshida S. Wakatake T. . (2018). Transcriptomic and metabolomic reprogramming from roots to haustoria in the parasitic plant, Thesium chinense. Plant Cell Physiol. 59, 724–733. doi: 10.1093/pcp/pcx200 29281058 PMC6018956

[B102] Ihl. B. Tutakhil. N. Hagen. A. Jacob. F. (1988). Studien an Cuscuta reflexa Roxb: VII. Zum Abwehrmechanismus von Lycopersicon esculentum Mill. Flora 181, 383–393. doi: 10.1016/s0367-2530(17)30380-8

[B103] Ishida J. K. Wakatake T. Yoshida S. Takebayashi Y. Kasahara H. Wafula E. . (2016). Local auxin biosynthesis mediated by a YUCCA flavin monooxygenase regulates haustorium development in the parasitic plant Phtheirospermum japonicum. Plant Cell 28, 1795–1814. doi: 10.1105/tpc.16.00310 27385817 PMC5006708

[B104] Janeway C. A. Jr. (1992). The immune system evolved to discriminate infectious nonself from noninfectious self. Immunol. Today 13, 11–16. doi: 10.1016/0167-5699(92)90198-g 1739426

[B105] Jeter C. R. Tang W. Henaff E. Butterfield T. Roux S. J. (2004). Evidence of a novel cell signaling role for extracellular adenosine triphosphates and diphosphates in Arabidopsis. Plant Cell 16, 2652–2664. doi: 10.1105/tpc.104.023945 15367717 PMC520962

[B106] Jhu M. Y. Ichihashi Y. Farhi M. Wong C. Sinha N. R. (2021). LATERAL ORGAN BOUNDARIES DOMAIN 25 functions as a key regulator of haustorium development in dodders. Plant Physiol. 186, 2093–2110. doi: 10.1093/plphys/kiab231 34618110 PMC8331169

[B107] Johnsen H. R. Striberny B. Olsen S. Vidal-Melgosa S. Fangel J. U. Willats W. G. . (2015). Cell wall composition profiling of parasitic giant dodder (Cuscuta reflexa) and its hosts: a priori differences and induced changes. New Phytol. 207, 805–816. doi: 10.1111/nph.13378 25808919

[B108] Kaga Y. Yokoyama R. Sano R. Ohtani M. Demura T. Kuroha T. . (2020). Interspecific signaling between the parasitic plant and the host plants regulate xylem vessel cell differentiation in haustoria of Cuscuta campestris. Front. Plant Sci. 11, 193. doi: 10.3389/fpls.2020.00193 32231674 PMC7082356

[B109] Kaiser B. Vogg G. Fürst U. B. Albert M. (2015). Parasitic plants of the genus Cuscuta and their interaction with susceptible and resistant host plants. Front. Plant Sci. 6. doi: 10.3389/fpls.2015.00045 25699071 PMC4316696

[B110] Kegge W. Ninkovic V. Glinwood R. Welschen R. A. M. Voesenek L. Pierik R. (2015). Red: far-red light conditions affect the emission of volatile organic compounds from barley (Hordeum vulgare), leading to altered biomass allocation in neighbouring plants. Ann. Bot. 115, 961–970. doi: 10.1093/aob/mcv120 25851141 PMC4407068

[B111] Kim G. Westwood J. H. (2015). Macromolecule exchange in Cuscuta-host plant interactions. Curr. Opin. Plant Biol. 26, 20–25. doi: 10.1016/j.pbi.2015.05.012 26051214

[B112] Kirschner G. K. Xiao T. T. Jamil M. Al-Babili S. Lube V. Blilou I. . (2023). A roadmap of haustorium morphogenesis in parasitic plants. J. Exp. Bot. 74, 7034–7044. doi: 10.1093/jxb/erad284 37486862 PMC10752351

[B113] Klosterman S. J. Chen J. Choi J. J. Chinn E. E. Hadwiger L. A. (2001). Characterization of a 20 kDa DNase elicitor from Fusarium solani f. sp. phaseoli and its expression at the onset of induced resistance in Pisum sativum. Mol. Plant Pathol. 2, 147–158. doi: 10.1046/j.1364-3703.2001.00062.x 20573002

[B114] Kohorn B. D. Johansen S. Shishido A. Todorova T. Martinez R. Defeo E. . (2009). Pectin activation of MAP kinase and gene expression is WAK2 dependent. Plant J. 60, 974–982. doi: 10.1111/j.1365-313x.2009.04016.x 19737363 PMC3575133

[B115] Kong C. H. Zhang S. Z. Li Y. H. Xia Z. C. Yang X. F. Meiners S. J. . (2018). Plant neighbor detection and allelochemical response are driven by root-secreted signaling chemicals. Nat. Commun. 9, 3867. doi: 10.1038/s41467-018-06429-1 30250243 PMC6155373

[B116] Kostoff D. (1928). Induced immunity in plants. Proc. Natl. Acad. Sci. U.S.A. 14, 236–237. doi: 10.1073/pnas.14.3.236 16587322 PMC1085447

[B117] Kubo M. Udagawa M. Nishikubo N. Horiguchi G. Yamaguchi M. Ito J. . (2005). Transcription switches for protoxylem and metaxylem vessel formation. Genes Dev. 19, 1855–1860. doi: 10.1101/gad.1331305 16103214 PMC1186185

[B118] Kucukoglu M. Nilsson J. Zheng B. Chaabouni S. Nilsson O. (2017). WUSCHEL-RELATED HOMEOBOX4 (WOX4)-like genes regulate cambial cell division activity and secondary growth in Populus trees. New Phytol. 215, 642–657. doi: 10.1111/nph.14631 28609015

[B43] Kuijt J. (1969). The biology of parasitic flowering plants. Berkeley, CA: University of California Press.

[B119] Kulkarni O. S. Mazumder M. Kini S. Hill E. D. Aow J. S. B. Phua S. M. L. . (2024). Volatile methyl jasmonate from roots triggers host-beneficial soil microbiome biofilms. Nat. Chem. Biol. 20, 473–483. doi: 10.1038/s41589-023-01462-8 37957272 PMC10972745

[B120] Kundariya H. Yang X. Morton K. Sanchez R. Axtell M. J. Hutton S. F. . (2020). MSH1-induced heritable enhanced growth vigor through grafting is associated with the RdDM pathway in plants. Nat. Commun. 11, 5343. doi: 10.1038/s41467-020-19140-x 33093443 PMC7582163

[B121] Kuromori T. Fujita M. Takahashi F. Yamaguchi-Shinozaki K. Shinozaki K. (2022). Inter-tissue and inter-organ signaling in drought stress response and phenotyping of drought tolerance. Plant J. 109, 342–358. doi: 10.1111/tpj.15619 34863007 PMC9300012

[B122] Kurotani K. Huang C. Okayasu K. Suzuki T. Ichihashi Y. Shirasu K. . (2022). Discovery of the interfamily grafting capacity of Petunia, a floricultural species. Hortic. Res. 9, uhab056. doi: 10.1093/hr/uhab056 35048114 PMC8969063

[B123] Kurotani K. I. Wakatake T. Ichihashi Y. Okayasu K. Sawai Y. Ogawa S. . (2020). Host-parasite tissue adhesion by a secreted type of β-1,4-glucanase in the parasitic plant Phtheirospermum japonicum. Commun. Biol. 3, 407. doi: 10.1038/s42003-020-01143-5 32733024 PMC7393376

[B124] Kutschmar A. Rzewuski G. Stührwohldt N. Beemster G. T. S. Inzé D. Sauter M. (2009). PSK-α promotes root growth in Arabidopsis. New Phytol. 181, 820–831. doi: 10.1111/j.1469-8137.2008.02710.x 19076296

[B125] La Malfa S. Bennici S. (2025). Genetics and molecular breeding of fruit tree species. Horticulturae 11, 1–154. doi: 10.3390/horticulturae1107075630654563

[B126] Laohavisit A. Wakatake T. Ishihama N. Mulvey H. Takizawa K. Suzuki T. . (2020). Quinone perception in plants via leucine-rich-repeat receptor-like kinases. Nature 587, 92–93. doi: 10.1038/s41586-020-2655-4 32879491

[B127] Lee C. Harvey J. T. Qin K. Joshi V. Leskovar D. I. (2024). Exploring the potential of Solanum pennellii and Solanum Peruvianum as rootstocks for enhancing thermotolerance of tomato plants. Environ. Exp. Bot. 221, 105741. doi: 10.1016/j.envexpbot.2024.10574142574925

[B128] Lev-Yadun S. Sprugel D. (2011). Why should trees have natural root grafts? Tree Physiol. 31, 575–578. doi: 10.1093/treephys/tpr061 21778291

[B132] Li W. Chu C. Li H. Zhang H. Sun H. Wang S. . (2024). Near-gapless and haplotype-resolved apple genomes provide insights into the genetic basis of rootstock-induced dwarfing. Nat. Genet. 56, 505–516. doi: 10.1038/s41588-024-01657-2 38347217

[B130] Li S. Han X. Yang L. Deng X. Wu H. Zhang M. . (2018). Mitogen-activated protein kinases and calcium-dependent protein kinases are involved in wounding-induced ethylene biosynthesis in Arabidopsis thaliana. Plant Cell Environ. 41, 134–147. doi: 10.1111/pce.12984 28543054

[B129] Li J. X. Timko M. P. (2009). Gene-for-gene resistance in Striga-cowpea associations. Science 325, 1094–1094. doi: 10.1126/science.1174754 19713520

[B131] Li S. Wang X. T. Xu W. Y. Liu T. Cai C. M. Chen L. Y. . (2021). Unidirectional movement of small RNAs from shoots to roots in interspecific heterografts. Nat. Plants 7, 50–59. doi: 10.1038/s41477-020-00829-2 33452489

[B134] Liu J. Abdelfattah A. Norelli J. Burchard E. Schena L. Droby S. . (2018). Apple endophytic microbiota of different rootstock/scion combinations suggests a genotype-specific influence. Microbiome 6. doi: 10.1186/s40168-018-0403-x 29374490 PMC5787276

[B135] Liu W. Fan J. Li J. Song Y. Li Q. Zhang Y. . (2014). SCF(SLF)-mediated cytosolic degradation of S-RNase is required for cross-pollen compatibility in S-RNase-based self-incompatibility in Petunia hybrida. Front. Genet. 5, 228. doi: 10.3389/fgene.2014.00228 25101113 PMC4106197

[B133] Liu C. Jia Y. He L. Li H. Song J. Ji L. . (2024). Integrated transcriptome and DNA methylome analysis reveal the biological base of increased resistance to gray leaf spot and growth inhibition in interspecific grafted tomato scions. BMC Plant Biol. 24, 130. doi: 10.1186/s12870-024-04764-8 38383283 PMC10880203

[B136] Lordan J. Zazurca L. Maldonado M. Torguet L. Alegre S. Miarnau X. (2019). Horticultural performance of 'Marinada' and 'Vairo' almond cultivars grown on a genetically diverse set of rootstocks. Sci. Hortic. 256. doi: 10.1016/j.scienta.2019.10855842574925

[B137] Lori M. van Verk M. C. Hander T. Schatowitz H. Klauser D. Flury P. . (2015). Evolutionary divergence of the plant elicitor peptides (Peps) and their receptors: interfamily incompatibility of perception but compatibility of downstream signalling. J. Exp. Bot. 66, 5315–5325. doi: 10.1093/jxb/erv236 26002971 PMC4526913

[B138] Loupit G. Brocard L. Ollat N. Cookson S. (2023). Grafting in plants: recent discoveries and new applications. J. Exp. Bot. 74, 2433–2447. doi: 10.1093/jxb/erad061 36846896

[B139] Lu D. P. Wu S. J. Gao X. Q. Zhang Y. L. Shan L. B. He P. (2010). A receptor-like cytoplasmic kinase, BIK1, associates with a flagellin receptor complex to initiate plant innate immunity. Proc. Natl. Acad. Sci. U.S.A. 107, 496–501. doi: 10.1073/pnas.0909705107 20018686 PMC2806711

[B140] Luu D. T. Qin X. Morse D. Cappadocia M. (2000). S-RNase uptake by compatible pollen tubes in gametophytic self-incompatibility. Nature 407, 649–651. doi: 10.1038/35036623 11034216

[B141] Macgregor S. R. Lee H. K. Nelles H. Johnson D. C. Zhang T. Ma C. . (2022). Autophagy is required for self-incompatible pollen rejection in two transgenic Arabidopsis thaliana accessions. Plant Physiol. 188, 2073–2084. doi: 10.1093/plphys/kiac026 35078230 PMC8969033

[B142] Mangeon A. Bell E. M. Lin W. C. Jablonska B. Springer P. S. (2011). Misregulation of the LOB domain gene DDA1 suggests possible functions in auxin signalling and photomorphogenesis. J. Exp. Bot. 62, 221–233. doi: 10.1093/jxb/erq259 20797997 PMC2993911

[B143] Marasco R. Rolli E. Fusi M. Michoud G. Daffonchio D. (2018). Grapevine rootstocks shape underground bacterial microbiome and networking but not potential functionality. Microbiome 6, 3. doi: 10.1186/s40168-017-0391-2 29298729 PMC5751889

[B144] Matsubayashi Y. Sakagami Y. (1996). Phytosulfokine, sulfated peptides that induce the proliferation of single mesophyll cells of Asparagus officinalis L. Proc. Natl. Acad. Sci. U.S.A. 93, 7623–7627. doi: 10.1073/pnas.93.15.7623 8755525 PMC38796

[B145] Matsuoka K. Sato R. Matsukura Y. Kawajiri Y. Iino H. Nozawa N. . (2021). Wound-inducible ANAC071 and ANAC096 transcription factors promote cambial cell formation in incised Arabidopsis flowering stems. Commun. Biol. 4, 369. doi: 10.1038/s42003-021-01895-8 33742091 PMC7979829

[B146] Matsuoka K. Sugawara E. Aoki R. Takuma K. Terao-Morita M. Satoh S. . (2016). Differential cellular control by cotyledon-derived phytohormones involved in graft reunion of Arabidopsis hypocotyls. Plant Cell Physiol. 57, 2620–2631. doi: 10.1093/pcp/pcw177 27986917

[B147] Matsuoka K. Yanagi R. Yumoto E. Yokota T. Yamane H. Satoh S. . (2018). RAP2.6L and jasmonic acid-responsive genes are expressed upon Arabidopsis hypocotyl grafting but are not needed for cell proliferation related to healing. Plant Mol. Biol. 96, 531–542. doi: 10.1007/s11103-018-0702-4 29344830

[B148] Mazumdar S. Augstein F. Zhang A. Musseau C. Anjam M. S. Marhavy P. . (2025). Damage activates EXG1 and RLP44 to suppress vascular differentiation during regeneration in Arabidopsis. Plant Commun. 6, 101256. doi: 10.1016/j.xplc.2025.101256 39818623 PMC12010363

[B149] Melnyk C. W. Gabel A. Hardcastle T. J. Robinson S. Miyashima S. Grosse I. . (2018). Transcriptome dynamics at Arabidopsis graft junctions reveal an intertissue recognition mechanism that activates vascular regeneration. Proc. Natl. Acad. Sci. U.S.A. 115, E2447–e2456. doi: 10.1073/pnas.1718263115 29440499 PMC5878008

[B150] Melnyk C. W. Schuster C. Leyser O. Meyerowitz E. M. (2015). A developmental framework for graft formation and vascular reconnection in *Arabidopsis thaliana*. Curr. Biol. 25, 1306–1318. doi: 10.1016/j.cub.2015.03.032 25891401 PMC4798781

[B151] Mng'omba S. du Toit E. Akinnifesi F. (2008). The relationship between graft incompatibility and phenols in *Uapaca kirkiana* Muell Arg. Sci. Hortic. 117, 212–218. doi: 10.1016/j.scienta.2008.03.031

[B152] Molnar A. Melnyk C. W. Bassett A. Hardcastle T. J. Dunn R. Baulcombe D. C. (2010). Small silencing RNAs in plants are mobile and direct epigenetic modification in recipient cells. Science 328, 872–875. doi: 10.1126/science.1187959 20413459

[B154] Moreno P. Ambrós S. Albiach-Martí M. R. Guerri J. Peña L. (2008). Citrus tristeza virus: a pathogen that changed the course of the citrus industry. Mol. Plant Pathol. 9, 251–268. doi: 10.1111/j.1364-3703.2007.00455.x 18705856 PMC6640355

[B153] Moreno M. A. Moreno M. A. Moing A. Lansac M. (1993). Peach/myrobalan plum graft incompatibility in the nursery. J. Hortic. Sci. 68, 705–714. doi: 10.1080/00221589.1993.1151640337339054

[B155] Mousavi S. A. Chauvin A. Pascaud F. Kellenberger S. Farmer E. E. (2013). *GLUTAMATE RECEPTOR-LIKE* genes mediate leaf-to-leaf wound signalling. Nature 500, 422–426. doi: 10.1038/nature12478 23969459

[B156] Mudge K. Janick J. Scofield S. Goldschmidt E. E. (2009). “ A history of grafting,” in Hortic Rev 33, 437–493. doi: 10.1002/9780470593776.ch9

[B157] Nawaz M. A. Imtiaz M. Kong Q. Cheng F. Ahmed W. Huang Y. . (2016). Grafting: a technique to modify Ion accumulation in horticultural crops. Front. Plant Sci. 7, 1457. doi: 10.3389/fpls.2016.01457 27818663 PMC5073839

[B158] Nishitani C. Demura T. Fukuda H. (2002). Analysis of early processes in wound-induced vascular regeneration using *TED3* and *ZeHB3* as molecular markers. Plant Cell Physiol. 43, 79–90. doi: 10.1093/pcp/pcf008 11828025

[B159] Nocito F. F. Espen L. Fedeli C. Lancilli C. Musacchi S. Serra S. . (2010). Oxidative stress and senescence-like status of pear calli co-cultured on suspensions of incompatible quince microcalli. Tree Physiol. 30, 450–458. doi: 10.1093/treephys/tpq006 20190345

[B160] Notaguchi M. Kurotani K. Sato Y. Tabata R. Kawakatsu Y. Okayasu K. . (2020). Cell-cell adhesion in plant grafting is facilitated by β-1,4-glucanases. Science 369, 698–69+. doi: 10.1126/science.abc3710 32764072

[B161] Olsen S. Striberny B. Hollmann J. Schwacke R. Popper Z. Krause K. (2016). Getting ready for host invasion: elevated expression and action of xyloglucan endotransglucosylases/hydrolases in developing haustoria of the holoparasitic angiosperm *Cuscuta*. J. Exp. Bot. 67, 695–708. doi: 10.1093/jxb/erv482 26561437 PMC4737069

[B162] Orozco-Cardenas M. Ryan C. A. (1999). Hydrogen peroxide is generated systemically in plant leaves by wounding and systemin via the octadecanoid pathway. Proc. Natl. Acad. Sci. U.S.A. 96, 6553–6557. doi: 10.1073/pnas.96.11.6553 10339626 PMC26920

[B163] Ota R. Ohkubo Y. Yamashita Y. Ogawa-Ohnishi M. Matsubayashi Y. (2020). Shoot-to-root mobile CEPD-like 2 integrates shoot nitrogen status to systemically regulate nitrate uptake in Arabidopsis. Nat. Commun. 11, 641. doi: 10.1038/s41467-020-14440-8 32005881 PMC6994653

[B164] Paajanen P. Tomkins M. Hoerbst F. Veevers R. Heeney M. Thomas H. . (2025). Re-analysis of mobile mRNA datasets raises questions about the extent of long-distance mRNA communication. Nat. Plants 11, 977–984. doi: 10.1038/s41477-025-01979-x 40240650 PMC12095074

[B165] Palauqui J. C. Elmayan T. Pollien J. M. Vaucheret H. (1997). Systemic acquired silencing: transgene-specific post-transcriptional silencing is transmitted by grafting from silenced stocks to non-silenced scions. EMBO J. 16, 4738–4745. doi: 10.1093/emboj/16.15.4738 9303318 PMC1170100

[B166] Park C. J. Ronald P. C. (2012). Cleavage and nuclear localization of the rice XA21 immune receptor. Nat. Commun. 3, 920. doi: 10.1038/ncomms1932 22735448 PMC3621455

[B167] Pearce G. Moura D. S. Stratmann J. Ryan C. A. Jr. (2001). RALF, a 5-kDa ubiquitous polypeptide in plants, arrests root growth and development. Proc. Natl. Acad. Sci. U.S.A. 98, 12843–12847. doi: 10.1073/pnas.201416998 11675511 PMC60141

[B168] Pearce G. Strydom D. Johnson S. Ryan C. A. (1991). A polypeptide from tomato leaves induces wound-inducible proteinase inhibitor proteins. Science 253, 895–897. doi: 10.1126/science.253.5022.895 17751827

[B169] Peters N. K. Frost J. W. Long S. R. (1986). A plant flavone, luteolin, induces expression of rhizobium meliloti nodulation genes. Science 233, 977–982. doi: 10.1126/science.3738520 3738520

[B170] Pham A. Q. Cho S. H. Nguyen C. T. Stacey G. (2020). *Arabidopsis* lectin receptor kinase P2K2 is a second plant receptor for extracellular ATP and contributes to innate immunity. Plant Physiol. 183, 1364–1375. doi: 10.1104/pp.19.01265 PMC7333714 32345768

[B171] Pieterse C. M. J. Zamioudis C. Berendsen R. L. Weller D. M. Van Wees S. C. M. Bakker P. (2014). Induced systemic resistance by beneficial microbes. Annu. Rev. Phytopathol. 52, 347–375. doi: 10.1146/annurev-phyto-082712-102340 24906124

[B172] Pina A. Zhebentyayeva T. Errea P. Abbott A. (2012). Isolation and molecular characterization of cinnamate 4-hydroxylase from apricot and plum. Biol. Plant 56, 441–450. doi: 10.1007/s10535-012-0114-230311153

[B173] Pitaksaringkarn W. Matsuoka K. Asahina M. Miura K. Sage-Ono K. Ono M. . (2014). XTH20 and XTH19 regulated by ANAC071 under auxin flow are involved in cell proliferation in incised *Arabidopsis* inflorescence stems. Plant J. 80, 604–614. doi: 10.1111/tpj.12654 25182467

[B174] Ranjan A. Ichihashi Y. Farhi M. Zumstein K. Townsley B. David-Schwartz R. . (2014). De novo assembly and characterization of the transcriptome of the parasitic weed dodder identifies genes associated with plant parasitism. Plant Physiol. 166, 1186–1199. doi: 10.1104/pp.113.234864 24399359 PMC4226353

[B175] Rasool A. Mansoor S. Bhat K. M. Hassan G. I. Baba T. R. AlYemeni M. N. . (2020). Mechanisms underlying graft union formation and rootstock-scion interaction in horticultural plants. Front. Plant Sci. 11. doi: 10.3389/fpls.2020.590847 33362818 PMC7758432

[B176] Reeves G. Tripathi A. Singh P. Jones M. R. W. Nanda A. K. Musseau C. . (2022). Monocotyledonous plants graft at the embryonic root-shoot interface. Nature 602, 280–286. doi: 10.1038/s41586-021-04247-y 34937943

[B177] Reig G. Salazar A. Zarrouk O. Forcada C. Val J. Moreno M. (2019). Long-term graft compatibility study of peach-almond hybrid and plum based rootstocks budded with European and Japanese plums. Sci. Hortic. 243, 392–400. doi: 10.1016/j.scienta.2018.08.03842574925

[B178] Reig G. Zarrouk O. Forcada C. Moreno M. (2018). Anatomical graft compatibility study between apricot cultivars and different plum based rootstocks. Sci. Hortic. 237, 67–73. doi: 10.1016/j.scienta.2018.03.03542574925

[B179] Rhodes J. Yang H. Moussu S. Boutrot F. Santiago J. Zipfel C. (2021). Perception of a divergent family of phytocytokines by the *Arabidopsis* receptor kinase MIK2. Nat. Commun. 12, 705. doi: 10.1038/s41467-021-20932-y 33514716 PMC7846792

[B180] Ruan Y. Dong S. Jiang S. Wang Y. Wu X. Zhuang Y. . (2026). The molecular basis of the binding and specific activation of rhizobial NodD by flavonoids. Science 391, 184–189. doi: 10.1126/science.aec3061 41505558

[B181] Runyon J. B. Mescher M. C. Felton G. W. De Moraes C. M. (2010). Parasitism by *Cuscuta* pentagona sequentially induces JA and SA defence pathways in tomato. Plant Cell Environ. 33, 290–303. doi: 10.1104/pp.107.112219 19930126

[B182] Samuel M. A. Chong Y. T. Haasen K. E. Aldea-Brydges M. G. Stone S. L. Goring D. R. (2009). Cellular pathways regulating responses to compatible and self-incompatible pollen in *Brassica* and Arabidopsis stigmas intersect at Exo70A1, a putative component of the exocyst complex. Plant Cell 21, 2655–2671. doi: 10.1105/tpc.109.069740 19789280 PMC2768929

[B183] Sanabria N. M. Huang J. C. Dubery I. A. (2010). Self/nonself perception in plants in innate immunity and defense. Self Nonself 1, 40–54. doi: 10.4161/self.1.1.10442 21559176 PMC3091600

[B184] Sánchez-Pérez R. Belmonte F. S. Borch J. Dicenta F. Moller B. L. Jorgensen K. (2012). Prunasin hydrolases during fruit development in sweet and bitter almonds. Plant Physiol. 158, 1916–1932. doi: 10.1104/pp.111.192021 PMC3320195 22353576

[B185] Sankaranarayanan S. Jamshed M. Samuel M. (2015). Degradation of glyoxalase I in *Brassica napus* stigma leads to self-incompatibility response. Nat. Plants 1, 15185. doi: 10.1038/nplants.2015.185 27251720

[B186] Scandola S. Samuel M. A. (2019). A flower-specific phospholipase D is a stigmatic compatibility factor targeted by the self-incompatibility response in *Brassica napus*. Curr. Biol. 29, 506–512.e504. doi: 10.1016/j.cub.2018.12.037 30661797

[B187] Schandry N. Becker C. (2020). Allelopathic plants: Models for studying plant-interkingdom interactions. Trends Plant Sci. 25, 176–185. doi: 10.1016/j.tplants.2019.11.004 31837955

[B188] Seemüller E. Schneider B. (2004). '*Candidatus* Phytoplasma Mali', '*Candidatus* Phytoplasma pyri' and '*Candidatus* Phytoplasma prunorum', the causal agents of apple proliferation, pear decline and European stone fruit yellows, respectively. Int. J. Syst. Evol. Microbiol. 54, 1217–1226. doi: 10.1099/ijs.0.02823-0 15280295

[B189] Sehr E. M. Agusti J. Lehner R. Farmer E. E. Schwarz M. Greb T. (2010). Analysis of secondary growth in the *Arabidopsis* shoot reveals a positive role of jasmonate signalling in cambium formation. Plant J. 63, 811–822. doi: 10.1111/j.1365-313x.2010.04283.x 20579310 PMC2988407

[B190] Serivichyaswat P. T. Kareem A. Feng M. Melnyk C. W. (2024). Auxin signaling in the cambium promotes tissue adhesion and vascular formation during *Arabidopsis* graft healing. Plant Physiol. 196, 754–762. doi: 10.1093/plphys/kiae257 38701036 PMC11444275

[B192] Shen Y. Xie Q. Wang T. Wang X. Xu F. Yan Z. . (2025). RALF33-FERONIA signaling orchestrates postwounding root-tip regeneration via TPR4-ERF115 dynamics. Plant Cell 37, koaf098. doi: 10.1093/plcell/koaf098 40323783 PMC12136156

[B191] Shen G. Zhang J. Lei Y. Xu Y. Wu J. (2023). Between-plant signaling. Annu. Rev. Plant Biol. 74, 367–386. doi: 10.1146/annurev-arplant-070122-015430 36626804

[B193] Shimizu K. Hozumi A. Aoki K. (2018). Organization of vascular cells in the haustorium of the parasitic flowering plant *Cuscuta japonica*. Plant Cell Physiol. 59, 715–723. doi: 10.1093/pcp/pcx197 29237029

[B194] Shu F. Liu F. Jin L. Chen G. Chen J. (2026). Bibliometric analysis of research on plant exosomes/extracellular vesicles: current status, hotspots, and trends. J. Holist Integr. Pharm. 7, 212–222. doi: 10.1016/j.jhip.2026.03.00542574925

[B195] Shu F. Wang D. Sarsaiya S. Jin L. Liu K. Zhao M. . (2024). Bulbil initiation: a comprehensive review on resources, development, and utilisation, with emphasis on molecular mechanisms, advanced technologies, and future prospects. Front. Plant Sci. 15. doi: 10.3389/fpls.2024.1343222 38650701 PMC11033377

[B196] Si T. Yang L. Lu J. Lin Y. Yu X. Zhang X. . (2025). Application of root exudates derived from peanut/maize intercropping system promotes peanut growth and yield via modulating nitrogen turnover processes. BMC Plant Biol. 25, 977. doi: 10.1186/s12870-025-06994-w 40731256 PMC12306142

[B197] Sicard A. Kappel C. Josephs E. B. Lee Y. W. Marona C. Stinchcombe J. R. . (2015). Divergent sorting of a balanced ancestral polymorphism underlies the establishment of gene-flow barriers in *Capsella*. Nat. Commun. 6. doi: 10.1038/ncomms8960 26268845 PMC4539569

[B198] Smet W. Sevilem I. de Luis Balaguer M. A. Wybouw B. Mor E. Miyashima S. . (2019). DOF2.1 controls cytokinin-dependent vascular cell proliferation downstream of TMO5/LHW. Curr. Biol. 29, 520–529.e526. doi: 10.1016/j.cub.2018.12.041 30686737 PMC6370950

[B199] Song W. Y. Wang G. L. Chen L. L. Kim H. S. Pi L. Y. Holsten T. . (1995). A receptor kinase-like protein encoded by the rice disease resistance gene, *Xa21*. Science 270, 1804–1806. doi: 10.1126/science.270.5243.1804 8525370

[B200] Sorty A. M. Kudjordjie E. N. Meena K. K. Nicolaisen M. Stougaard P. (2025). Plant root exudates: advances in belowground signaling networks, resilience, and ecosystem functioning for sustainable agriculture. Plant Stress 17, 100907. doi: 10.1016/j.stress.2025.10090742574925

[B201] Soto-Cruz F. Zorrilla J. Rial C. Varela R. Molinillo J. Igartuburu J. . (2021). Allelopathic activity of strigolactones on the germination of parasitic plants and arbuscular mycorrhizal fungi growth. Agron. 11. doi: 10.3390/agronomy1111217430654563

[B202] Stegemann S. Bock R. (2009). Exchange of genetic material between cells in plant tissue grafts. Science 324, 649–651. doi: 10.1126/science.1170397 19407205

[B203] Stegmann M. Monaghan J. Smakowska-Luzan E. Rovenich H. Lehner A. Holton N. . (2017). The receptor kinase FER is a RALF-regulated scaffold controlling plant immune signaling. Science 355, 287–289. doi: 10.1126/science.aal2541 28104890

[B204] Steinauer K. Chatzinotas A. Eisenhauer N. (2016). Root exudate cocktails: the link between plant diversity and soil microorganisms? Ecol. Evol. 6, 7387–7396. doi: 10.1002/ece3.2454 28725406 PMC5513276

[B205] Stirnemann E. M. Sasse J. (2025). How to harness the effects of exudates and microbes that support beneficial plant-plant interactions for sustainable agriculture. PloS Biol. 23, e3003416. doi: 10.1371/journal.pbio.3003416 41100520 PMC12530572

[B206] Stringlis I. A. Yu K. Feussner K. de Jonge R. van Bentum S. Van Verk M. C. . (2018). MYB72-dependent coumarin exudation shapes root microbiome assembly to promote plant health. Proc. Natl. Acad. Sci. U.S.A. 115, E5213–E5222. doi: 10.1073/pnas.1722335115 29686086 PMC5984513

[B207] Su C. Liu H. Wafula E. K. Honaas L. de Pamphilis C. W. Timko M. P. (2020). SHR4z, a novel decoy effector from the haustorium of the parasitic weed *Striga gesnerioides*, suppresses host plant immunity. New Phytol. 226, 891–908. doi: 10.1111/nph.16351 31788811 PMC7187149

[B208] Takahashi F. Suzuki T. Osakabe Y. Betsuyaku S. Kondo Y. Dohmae N. . (2018). A small peptide modulates stomatal control via abscisic acid in long-distance signalling. Nature 556, 235–23+. doi: 10.1038/s41586-018-0009-2 29618812

[B209] Takasaki T. Hatakeyama K. Suzuki G. Watanabe M. Isogai A. Hinata K. (2000). The S receptor kinase determines self-incompatibility in *Brassica stigma*. Nature 403, 913–916. doi: 10.1038/35002628 10706292

[B210] Takayama S. Isogai A. (2005). Self-incompatibility in plants. Annu. Rev. Plant Biol. 56, 467–489. doi: 10.1146/annurev.arplant.56.032604.144249 15862104

[B211] Takayama S. Shiba H. Iwano M. Shimosato H. Che F. S. Kai N. . (2000). The pollen determinant of self-incompatibility in *Brassica campestris*. Proc. Natl. Acad. Sci. U.S.A. 97, 1920–1925. doi: 10.1073/pnas.040556397 10677556 PMC26537

[B212] Takei S. Uchiyama Y. Bürger M. Suzuki T. Okabe S. Chory J. . (2023). A divergent clade KAI2 protein in the root parasitic plant *Orobanche* minor is a highly sensitive strigolactone receptor and is involved in the perception of sesquiterpene lactones. Plant Cell Physiol. 64, 996–1007. doi: 10.1093/pcp/pcad026 37061839 PMC10504577

[B213] Tan T. T. Endo H. Sano R. Kurata T. Yamaguchi M. Ohtani M. . (2018). Transcription factors VND1-VND3 contribute to cotyledon xylem vessel formation. Plant Physiol. 176, 773–789. doi: 10.1104/pp.17.00461 29133368 PMC5761765

[B214] Tanaka K. Choi J. Cao Y. Stacey G. (2014). Extracellular ATP acts as a damage-associated molecular pattern (DAMP) signal in plants. Front. Plant Sci. 5, 446. doi: 10.3389/fpls.2014.00446 25232361 PMC4153020

[B215] Tanaka K. Heil M. (2021). Damage-associated molecular patterns (DAMPs) in plant innate immunity: applying the danger model and evolutionary perspectives. Annu. Rev. Phytopathol. 59, 53–75. doi: 10.1146/annurev-phyto-082718-100146 33900789

[B216] Tedesco S. Irisarri P. Teixeira Santos M. Fevereiro P. Pina A. Kragler F. (2023). Early detection of grapevine graft incompatibility: insights into translocated and virus-induced incompatibility. Sci. Hortic. 318, 112087. doi: 10.1016/j.scienta.2023.11208742574925

[B217] Thieme C. J. Rojas-Triana M. Stecyk E. Schudoma C. Zhang W. N. Yang L. . (2015). Endogenous *Arabidopsis* messenger RNAs transported to distant tissues. Nat. Plants 1, 15025. doi: 10.1007/s00427-003-0298-8 27247031

[B218] Thomas H. R. Gevorgyan A. Frank M. H. (2023). Anatomical and biophysical basis for graft incompatibility within the *Solanaceae*. J. Exp. Bot. 74, 4461–4470. doi: 10.1093/jxb/erad155 37103969 PMC10687351

[B219] Thomas H. R. Gevorgyan A. Hermanson A. Yanders S. Erndwein L. Norman-Ariztía M. . (2024). Graft incompatibility between pepper and tomato elicits an immune response and triggers localized cell death. Hortic. Res. 11, uhae255. doi: 10.1093/hr/uhae255 39664688 PMC11630344

[B220] Thomas H. R. Van den Broeck L. Spurney R. Sozzani R. Frank M. (2022). Gene regulatory networks for compatible versus incompatible grafts identify a role for SlWOX4 during junction formation. Plant Cell 34, 535–556. doi: 10.1093/plcell/koab246 34609518 PMC8846177

[B221] Timko M. P. Gowda B. S. Ouedraogo J. Ousmane B. (2007). Integrating New Technologies for Striga Control (Singapore: World Scientific).

[B222] Toh S. Inoue S. Toda Y. Yuki T. Suzuki K. Hamamoto S. . (2018). Identification and characterization of compounds that affect stomatal movements. Plant Cell Physiol. 59, 1568–1580. doi: 10.1093/pcp/pcy061 29635388

[B223] Tomaz Z. F. P. Rodrigues A. C. Veríssimo V. Marafon A. C. Herter F. G. Rufato A. D. (2009). Compatibilidade de enxertia de cultivares de marmeleiros compereiras. Rev. Bras. Frutic 31, 1211–1217. doi: 10.1590/s0100-2945200900040004141099703

[B224] Tomilov A. A. Tomilova N. B. Abdallah I. Yoder J. I. (2005). Localized hormone fluxes and early haustorium development in the hemiparasitic plant *Triphysaria versicolor*. Plant Physiol. 138, 1469–1480. doi: 10.1104/pp.104.057836 15965023 PMC1176418

[B225] Toyota M. Spencer D. Sawai-Toyota S. Jiaqi W. Zhang T. Koo A. J. . (2018). Glutamate triggers long-distance, calcium-based plant defense signaling. Science 361, 1112–1115. doi: 10.1126/science.aat7744 30213912

[B226] Turnbull C. G. Booker J. P. Leyser H. M. (2002). Micrografting techniques for testing long-distance signalling in *Arabidopsis*. Plant J. 32, 255–262. doi: 10.1046/j.1365-313X.2002.01419.x 12383090

[B227] Venema J. H. Dijk B. E. Bax J. M. van Hasselt P. R. Elzenga J. T. M. (2008). Grafting tomato (*Solanum lycopersicum*) onto the rootstock of a high-altitude accession of *Solanum habrochaites* improves suboptimal-temperature tolerance. Environ. Exp. Bot. 63, 359–367. doi: 10.1016/j.envexpbot.2007.12.01542574925

[B228] Wakatake T. Yoshida S. Shirasu K. (2018). Induced cell fate transitions at multiple cell layers configure haustorium development in parasitic plants. Development 145, dev164848. doi: 10.1242/dev.164848 29950390 PMC6078332

[B229] Walters D. R. Ratsep J. Havis N. D. (2013). Controlling crop diseases using induced resistance: challenges for the future. J. Exp. Bot. 64, 1263–1280. doi: 10.1093/jxb/ert026 23386685

[B234] Wang L. Einig E. Almeida-Trapp M. Albert M. Fliegmann J. Mithöfer A. . (2018). The systemin receptor SYR1 enhances resistance of tomato against herbivorous insects. Nat. Plants 4, 152–156. doi: 10.1038/s41477-018-0106-0 29459726

[B231] Wang G. Hu C. Zhou J. Liu Y. Cai J. Pan C. . (2019a). Systemic root-shoot signaling drives Jasmonate-based root defense against *Nematodes*. Curr. Biol. 29, 3430–3438.e3434. doi: 10.1016/j.cub.2019.08.049 31588001

[B233] Wang J. Jin Z. Yin H. Yan B. Ren Z. Xu J. . (2014). Auxin redistribution and shifts in *PIN* gene expression during *Arabidopsis* grafting. Russ. J. Plant Physiol. 61, 688–696. doi: 10.1134/s102144371405015x

[B235] Wang L. Lin Z. Carli J. Gladala-Kostarz A. Davies J. Franklin-Tong V. . (2022). ATP depletion plays a pivotal role in self-incompatibility, revealing a link between cellular energy status, cytosolic acidification and actin remodelling in pollen tubes. New Phytol. 236, 1691–1707. doi: 10.1111/nph.18350 35775998 PMC9796540

[B230] Wang D. Wei L. Liu T. Ma J. Huang K. Guo H. . (2023). Suppression of ETI by PTI priming to balance plant growth and defense through an MPK3/MPK6-WRKYs-PP2Cs module. Mol. Plant 16, 903–918. doi: 10.1016/j.molp.2023.04.004 37041748

[B232] Wang H. Zhou P. Zhu W. Wang F. (2019b). De novo comparative transcriptome analysis of genes differentially expressed in the scion of homografted and heterografted tomato seedlings. Sci. Rep. 9, 20240. doi: 10.1038/s41598-019-56563-z 31882801 PMC6934607

[B236] Weiberg A. Wang M. Lin F. M. Zhao H. W. Zhang Z. H. Kaloshian I. . (2013). Fungal small RNAs suppress plant immunity by hijacking host RNA interference pathways. Science 342, 118–123. doi: 10.1126/science.1239705 24092744 PMC4096153

[B237] Wheeler M. J. de Graaf B. H. Hadjiosif N. Perry R. M. Poulter N. S. Osman K. . (2009). Identification of the pollen self-incompatibility determinant in *Papaver rhoeas*. Nature 459, 992–995. doi: 10.1038/nature08027 19483678 PMC2699350

[B238] Wheeler M. J. Vatovec S. Franklin-Tong V. E. (2010). The pollen S-determinant in Papaver: comparisons with known plant receptors and protein ligand partners. J. Exp. Bot. 61, 2015–2025. doi: 10.1093/jxb/erp383 20097844

[B239] Williams B. Ahsan M. U. Frank M. H. (2021). Getting to the root of grafting-induced traits. Curr. Opin. Plant Biol. 59, 101988. doi: 10.1016/j.pbi.2020.101988 33388626

[B240] Wu Y. Gao Y. Zhan Y. Kui H. Liu H. Yan L. . (2020). Loss of the common immune coreceptor BAK1 leads to NLR-dependent cell death. Proc. Natl. Acad. Sci. U.S.A. 117, 27044–27053. doi: 10.1073/pnas.1915339117 33055218 PMC7604517

[B241] Wu Y. M. Ma Y. J. Wang M. Zhou H. Gan Z. M. Zeng R. F. . (2022). Mobility of FLOWERING LOCUS T protein as a systemic signal in trifoliate orange and its low accumulation in grafted juvenile scions. Hortic. Res. 9, uhac056. doi: 10.1093/hr/uhac056 35702366 PMC9186307

[B242] Wulf K. E. Reid J. B. Foo E. (2020). What drives interspecies graft union success? Exploring the role of phylogenetic relatedness and stem anatomy. Physiol. Plant 170, 132–147. doi: 10.1111/ppl.13118 32385889

[B243] Xiong M. Liu C. Guo L. Wang J. Wu X. Li L. . (2021). Compatibility evaluation and anatomical observation of melon grafted onto eight cucurbitaceae species. Front. Plant Sci. 12. doi: 10.3389/fpls.2021.762889 34745194 PMC8563831

[B244] Xiong M. Zhang T. Qian X. Kadeer A. Kurotani K. I. Li L. . (2026). *Xyloglucan endotransglucosylase*/*hydrolase* family genes are required for the plant graft union formation through callus proliferation. Plant Physiol. 200, kiag030. doi: 10.1093/plphys/kiag030 41612937

[B245] Yamada T. MArubashi W. (2003). Overproduced ethylene causes programmed cell death leading to temperature-sensitive lethality in hybrid seedlings from the cross *Nicotiana suaveolens* x *N-tabacum*. Planta 217, 690–698. doi: 10.1007/s00425-003-1035-2 12728318

[B246] Yamaguchi Y. Huffaker A. Bryan A. C. Tax F. E. Ryan C. A. (2010). PEPR2 is a second receptor for the Pep1 and Pep2 peptides and contributes to defense responses in *Arabidopsis*. Plant Cell 22, 508–522. doi: 10.1105/tpc.109.068874 20179141 PMC2845411

[B247] Yamaguchi Y. Pearce G. Ryan C. A. (2006). The cell surface leucine-rich repeat receptor for AtPep1, an endogenous peptide elicitor in *Arabidopsis*, is functional in transgenic tobacco cells. Proc. Natl. Acad. Sci. U.S.A. 103, 10104–10109. doi: 10.1073/pnas.0603729103 16785433 PMC1502513

[B248] Yamamoto E. Takashi T. Morinaka Y. Lin S. Y. Wu J. Z. Matsumoto T. . (2010). Gain of deleterious function causes an autoimmune response and Bateson-Dobzhansky-Muller incompatibility in rice. Mol. Genet. Genomics 283, 305–315. doi: 10.1007/s00438-010-0514-y 20140455

[B249] Yan J. Zhang C. Gu M. Bai Z. Zhang W. Qi T. . (2009). The Arabidopsis CORONATINE INSENSITIVE1 protein is a jasmonate receptor. Plant Cell 21, 2220–2236. doi: 10.1105/tpc.109.065730 19717617 PMC2751961

[B251] Yang L. Chen Y. Liu X. J. Zhang S. Han Q. Q. (2023a). Genome-wide identification and expression analysis of xyloglucan endotransglucosylase/hydrolase genes family in *Salicaceae* during grafting. BMC Genomics 24, 676. doi: 10.1186/s12864-023-09762-y 37946112 PMC10636897

[B252] Yang L. Machin F. Wang S. F. Saplaoura E. Kragler F. (2023b). Heritable transgene-free genome editing in plants by grafting of wild-type shoots to transgenic donor rootstocks. Nat. Biotechnol. 41, 958–967. doi: 10.1038/s41587-022-01585-8 36593415 PMC10344777

[B250] Yang H. Matsubayashi Y. Hanai H. Sakagami Y. (2000). Phytosulfokine-alpha, a peptide growth factor found in higher plants: its structure, functions, precursor and receptors. Plant Cell Physiol. 41, 825–830. doi: 10.1093/pcp/pcd009 10965938

[B254] Yang Z. Wafula E. K. Honaas L. A. Zhang H. Das M. Fernandez-Aparicio M. . (2015). Comparative transcriptome analyses reveal core parasitism genes and suggest gene duplication and repurposing as sources of structural novelty. Mol. Biol. Evol. 32, 767–790. doi: 10.1093/molbev/msu343 25534030 PMC4327159

[B253] Yang L. Xia L. Zeng Y. Han Q. Zhang S. (2022). Grafting enhances plants drought resistance: Current understanding, mechanisms, and future perspectives. Front. Plant Sci. 13, 1015317. doi: 10.3389/fpls.2022.1015317 36275555 PMC9583147

[B255] Yeoman M. M. (1984). “ Cellular recognition systems in grafting,” in Cellular Interactions. Eds. Linskens H. F. Heslop-Harrison J. ( Springer Berlin Heidelberg, Berlin, Heidelberg), 453–472.

[B256] Yoneyama K. Awad A. A. Xie X. Yoneyama K. Takeuchi Y. (2010). Strigolactones as germination stimulants for root parasitic plants. Plant Cell Physiol. 51, 1095–1103. doi: 10.1093/pcp/pcq055 20403809 PMC2900819

[B257] Yoo B. Kragler F. Varkonyi-Gasic E. Haywood V. Archer-Evans S. Lee Y. . (2004). A systemic small RNA signaling system in plants. Plant Cell 16, 1979–2000. doi: 10.1105/tpc.104.023614 15258266 PMC519190

[B258] Yu X. Q. Niu H. Q. Liu C. Wang H. L. Yin W. Xia X. (2024). PTI-ETI synergistic signal mechanisms in plant immunity. Plant Biotechnol. J. 22, 2113–2128. doi: 10.1111/pbi.14332 38470397 PMC11258992

[B259] Zarrouk O. Testillano P. Risueño M. Moreno M. Gogorcena Y. (2010). Changes in cell/tissue organization and peroxidase activity as markers for early detection of graft incompatibility in peach/plum combinations. J. Am. Soc Hortic. Sci. 135, 9–17. doi: 10.21273/jashs.135.1.9

[B260] Zeidler D. Dubery I. A. Schmitt-Kopplin P. Von Rad U. Durner J. (2010). Lipopolysaccharide mobility in leaf tissue of *Arabidopsis thaliana*. Mol. Plant Pathol. 11, 747–755. doi: 10.1111/j.1364-3703.2010.00638.x 21029320 PMC6640497

[B261] Zhai L. Wang X. Tang D. Qi Q. Yer H. Jiang X. . (2021). Molecular and physiological characterization of the effects of auxin-enriched rootstock on grafting. Hortic. Res. 8, 74. doi: 10.1038/s41438-021-00509-y 33790234 PMC8012700

[B262] Zhan C. Xue N. Su Z. Zheng T. Wu J. (2025). RBOHD, GLR3.3, and GLR3.6 cooperatively control wounding hypocotyl-induced systemic Ca^2+^ signals, jasmonic acid, and glucosinolates in *Arabidopsis* leaves. Plant Divers. 47, 690–701. doi: 10.1016/j.pld.2025.05.004 40734840 PMC12302501

[B265] Zhang H. Hu Z. Lei C. Zheng C. Wang J. Shao S. . (2018). A plant phytosulfokine peptide initiates auxin-dependent immunity through cytosolic Ca^2+^ signaling in tomato. Plant Cell 30, 652–667. doi: 10.1105/tpc.17.00537 29511053 PMC5894845

[B266] Zhang W. N. Kollwig G. Stecyk E. Apelt F. Dirks R. Kragler F. (2014). Graft-transmissible movement of inverted-repeat-induced siRNA signals into flowers. Plant J. 80, 106–121. doi: 10.1111/tpj.12622 25039964

[B263] Zhang A. Matsuoka K. Kareem A. Robert M. Roszak P. Blob B. . (2022). Cell-wall damage activates DOF transcription factors to promote wound healing and tissue regeneration in *Arabidopsis thaliana*. Curr. Biol. 32, 1883–1894.e1887. doi: 10.1016/j.cub.2022.02.069 35320706

[B264] Zhang A. J. Wang T. J. Yuan L. Shen Y. X. Liu K. Liu B. . (2024). Horizontal transfer of plasmid-like extrachromosomal circular DNAs across graft junctions in *Solanaceae*. Mol. Genet. Genomics 4. doi: 10.1186/s43897-024-00124-0 39563413 PMC11577957

[B267] Zhang X. Yang Z. Wu D. Yu F. (2020). RALF-FERONIA signaling: linking plant immune response with cell growth. Plant Commun. 1, 100084. doi: 10.1016/j.xplc.2020.100084 33367248 PMC7747976

[B268] Zhu Y. Hu S. Min J. Zhao Y. Yu H. Irfan M. . (2024). Transcriptomic analysis provides an insight into the function of CmGH9B3, a key gene of β-1, 4-glucanase, during the graft union healing of oriental melon scion grafted onto squash rootstock. Biotechnol. J. 19, 2400006. doi: 10.1002/biot.202400006 38581090

